# Implantable Multifunctional Micro‐Oxygen Reservoir System for Promoting Vascular‐Osteogenesis via Remodeling Regenerative Microenvironment

**DOI:** 10.1002/advs.202409636

**Published:** 2024-11-26

**Authors:** Min Rui, Jiannan Mao, Hongshuai Wu, Yujian Hui, Hao Shen, Yilin Yang, Tao Ma, Kewei Ren, Juan Wang, Wenguo Cui, Qin Shi, Huilin Yang

**Affiliations:** ^1^ Department of Orthopaedics The First Affiliated Hospital of Soochow University Orthopaedic Institute of Soochow University 899 Pinghai Road Suzhou Jiangsu 215031 P. R. China; ^2^ Department of Orthopaedics Wuxi Key Laboratory of Biomaterials for Clinical Application Department of Central Laboratory Jiangyin Clinical College of Xuzhou Medical University No.163 Shoushan Road Jiangyin Jiangsu 214400 P. R. China; ^3^ Department of Orthopedics The First Affiliated Hospital of Wannan Medical College Yijishan Hospital, No. 2, Zhe Shan Xi Road Wuhu Anhui 241001 P. R. China; ^4^ Department of Orthopaedics Shanghai Key Laboratory for Prevention and Treatment of Bone and Joint Diseases Shanghai Institute of Traumatology and Orthopaedics Ruijin Hospital Shanghai Jiao Tong University School of Medicine 197 Ruijin 2nd Road Shanghai 200025 P. R. China

**Keywords:** bone regeneration, hydrogel microsphere, hypoxia, microenvironment regulation, oxygen generation, reactive oxygen species

## Abstract

Hypoxia and reactive oxygen species (ROS) overaccumulation cause persistent oxidative stress and impair intrinsic regenerative potential upon tissue injury. For local tissue injury with hypoxia, such as bone fracture and defects, a localized‐sufficient oxygen supply is highly desirable but remains challenging. Therefore, to explore a strategy and its intrinsic mechanism for supplying oxygen locally and remodeling the regenerative microenvironment, an innovative oxygenating hydrogel microsphere system with sustained oxygenation and antioxidant properties is introduced by loading CaO_2_@SiO_2_@PDA (CSP) nanoparticles. Specifically, the CSP nanoparticles exhibited broad‐spectrum free radicals scavenging ability, along with prolonged controlled‐release of oxygen once integrated into the gelatin methacrylate anhydride (GelMA) microspheres (CSP‐GM). The CSP‐GM with extra cellular matrix (ECM)‐mimicking structures reconstructed living niches, promoting the adhesion and proliferation of bone marrow stromal cells (BMSCs). As a multifaceted microenvironment regulator, CSP‐GM remodeled the regenerative microenvironment by synergistically producing oxygen and scavenging ROS, recovering mitochondrial homeostasis and antioxidant defenses of BMSCs, promoting angiogenesis and osteogenesis under hypoxia conditions via precisely modulating the Nrf2/HO‐1 signaling pathway. The multiple pro‐regenerative effects of the implantable functionalized micro‐oxygen reservoir on bone repair are further corroborated by the enhanced vascularized bone formation in rat femoral defects, presenting a comprehensive and promising strategy for tissue repair.

## Graphical abstract

An oxygenating hydrogel microsphere (CSP‐GM) integrated with CSP nanoparticles is developed as a multifaceted microenvironment regulator. This innovative system effectively remodels the regenerative microenvironment by synergistically producing oxygen and scavenging ROS, recovering mitochondrial homeostasis and antioxidant defenses of BMSCs, promoting angiogenesis and osteogenesis under hypoxia conditions via precisely modulating the Nrf2/HO‐1 signaling pathways, thereby facilitating bone regeneration.

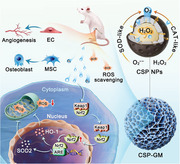

## 1 Introduction

With the acceleration of aging population and the increasing prevalence of accidents worldwide, the incidence of bone fractures is rising annually. Bone defects and impaired fracture‐unions, which are among the most common severe complications of fractures, not only impose substantial economic and social burdens but also greatly compromise patients' quality of life.^[^ ^1^ ^]^ Regrettably, achieving optimal bone repair for fractures and large bone defects remains a formidable challenge. The regeneration of damaged bone tissue is a complex and dynamic process, involving intricate interactions between cells and their microenvironment. Following bone fractures, impaired vasculature impedes oxygen delivery to the bone defect areas, creating a hypoxic microenvironment around the affected regions.^[^ ^2^ ^]^ The adverse hypoxic microenvironment not only inhibits the proliferation and differentiation of mesenchymal stem/stromal cells (MSCs), but also impairs the aerobic metabolism and functionality of reparative cells engaged in bone regeneration.^[^ ^2^, ^3^ ^]^ Thereby the expression of osteogenesis‐specific genes is significantly down‐regulated, and collagen type I synthesis and mineralization progress are also negatively affected.^[^ ^4^ ^]^ Over recent decades, various innovative biomaterials endowed with specific bio‐functions have been designed in the field of bone tissue engineering, providing new avenues for the treatment of bone defects. Nevertheless, their therapeutic efficacy remains limited, as they fail to effectively rehabilitate the impaired regenerative microenvironment of bone defects.^[^ ^5^ ^]^ Ideal biomaterials should not facilitate the integration between material and cells, but also promote endogenous repair and the regeneration of damaged bone tissues by regulating the disordered microenvironment.

Previous studies have demonstrated the efficacy of oxygenation in promoting fracture healing, and hyperbaric oxygen therapy is recommended as an adjunctive treatment for cases of traumatic bone ischemia and nonunion.^[^ ^6^ ^]^ However, as a systemic oxygen delivery strategy, it fails to continuously provide sufficient oxygen to hypoxic areas and carries the potential risk of oxygen toxicity.^[^ ^7^ ^]^ To address hypoxia within the tissue microenvironment, various topical oxygenating systems had been devised, including perfluorocarbons (PFCs),^[^ ^4b^ ^]^ catalase (CAT)‐mimicking nanozymes^[^ ^8^ ^]^ and solid inorganic peroxides.^[^ ^9^ ^]^ However, these systems still faced unresolved challenges in practical application. Specifically, as oxygen carriers, the oxygen supply sustainability of PFCs was markedly compromised in oxygen deprivation environments. Moreover, the oxygen supply was inadequate by utilizing CAT‐mimicking nanoparticles to convert endogenous hydrogen peroxide to oxygen at the injury site.^[^ ^8^ ^]^ Their poor oxygen production capacity made them less effective in alleviating prolonged hypoxia during the bone generation phase.^[^ ^4^, ^10^ ^]^ Currently, calcium peroxide (CPO) has attracted considerable attention in the field of tissue engineering owing to its remarkable oxygen production performance.^[^ ^9^, ^11^ ^]^ The primary concern associated with utilizing CPO was its limited controllability in oxygen release, causing unpredictable kinetics and a potentially excessive accumulation of hydrogen peroxide, both of which were undesirable for bone repair.^[^ ^9^, ^11^ ^]^ Encapsulating CPO with hydrophobic barriers had been demonstrated as a feasible approach to enhance stability and optimize oxygen release, thereby improving their biological security.^[^ ^11^, ^12^ ^]^ Additionally, a separate study revealed that silica shell barriers could significantly attenuate the diffusion of gas molecules released from internal nanoparticles, effectively prolonging their release and enabling precise control over the reaction process.^[^ ^13^ ^]^ Similarly, encapsulating CPO within silica shell ensures stable and sustained oxygen generation, while concurrently minimizing potential cytotoxic effects, which meets the oxygen supply requirements for tissue regeneration in bone defects. Moreover, it has been acknowledged that Ca^2+^ and silicate can promote osteogenesis, and expertly incorporating these bioactive elements into oxygenating biomaterials, thereby conducing to bone regeneration.^[^ ^14^ ^]^

Increasing evidence indicated that hypoxia contributes to the overproduction of reactive oxygen species (ROS), disrupting ROS homeostasis and triggering oxidative stress.^[^ ^4^, ^15^ ^]^ Oxidative damage can lead to mitochondrial dysfunction, deoxyribonucleic acid (DNA), and protein damage, which ultimately resulted in extensive apoptosis of osteoblasts and inhibition on osteogenic differentiation of BMSCs.^[^ ^4^, ^8^, ^16^ ^]^ In turn, the excessive ROS not only impeded angiogenesis but also consumed localized oxygen, potentially exacerbating hypoxia and establishing a vicious circle.^[^ ^15^, ^17^ ^]^ In previous studies, CAT was incorporated into oxygen‐releasing hydrogel endowed it with ROS‐scavenging ability, which allowed the hydrogel to alleviate hypoxia while reversing oxidative stress.^[^ ^4^, ^7^ ^]^ Unfortunately, the poor stability and diminished antioxidant efficiency of natural enzymes under adverse conditions (e.g., redox imbalance or acidic environment) were ignored.^[^ ^18^ ^]^ Polydopamine (PDA), a natural biopolymer, had demonstrated remarkable ROS scavenging effects, featuring a broad‐spectrum free radical neutralization capacity and high stability in physiological conditions.^[^ ^19^ ^]^ Moreover, owing to its superior biocompatibility, biodegradability, extraordinary adhesive properties, and gentle synthesis requirements, PDA has emerged as a promising candidate for the surface functionalization of nanoparticles.^[^ ^16^, ^18^, ^19^, ^20^ ^]^ Meanwhile, the integration of PDA coating with superoxide dismutase and catalase mimetic activities, can also effectively decompose the excess hydrogen peroxide generated in the reaction of CaO_2_.^[^ ^19a^ ^]^ Drawing on these findings, it is feasible to design bio‐compatible nanocomposites that possess favorable antioxidative and oxygen supply properties.

The enhancement of bio‐function and achievement of nanoparticles enrichment, along with their long‐term retention at the injury site, should also be considered. A suitable carrier is crucial for the efficient encapsulation and targeted release of nanoparticles. Gelatin methacrylate anhydride (GelMA), a biomaterial noted for its excellent biocompatibility, can be fabricated into uniform hydrogel microspheres by microfluidic technology.^[^ ^21^ ^]^ Moreover, the amine groups present in PDA coatings facilitated the effective grafting of nanoparticles to GelMA microspheres.^[^ ^21b^ ^]^ Serving as superior carriers for nanoparticles, micro‐nano hydrogel microspheres can offer an optimal living niche for reparative cells and effectively ameliorate the adverse microenvironment via local delivery.^[^ ^21^, ^22^ ^]^ Therefore, this multifunctional platform could synergistically integrate the respective advantages of nanozymes and hydrogels to optimize therapeutic efficacy.

**Scheme 1:**
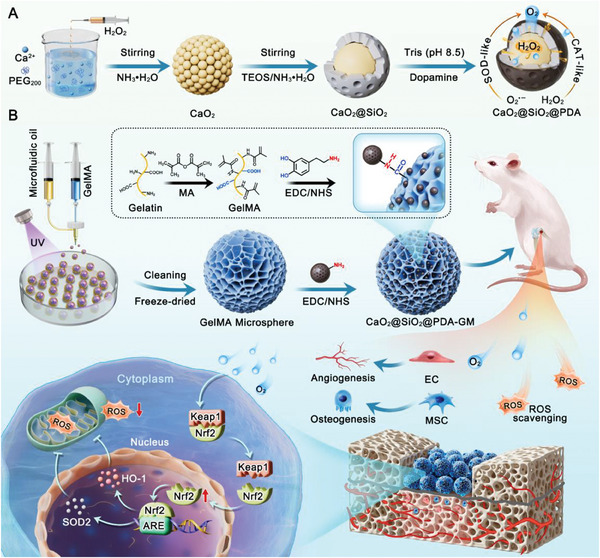
Schematic illustration of composite oxygenating microspheres for promoting vascularized bone regeneration with multi‐hierarchy bone microenvironment regulation. A) Fabrication of composite CSP oxygenating nanoparticles with ROS‐scavenging enzymes activities. B) Preparation of functionalized GelMA hydrogel microspheres (CSP‐GM) via covalent grafting of CSP nanoparticles. CSP‐GM hydrogel microspheres facilitate bone repair through dual activation of vascular‐osteogenesis and local microenvironment regulation for critical femoral condylar defect in rats.

In this study, following the concept that the hyperbaric oxygen chamber is widely applied to treat diseases in the clinic, we initially demonstrated the essentiality of oxygen supply and ROS scavenging in remodeling the functionality of BMSCs within a hypoxic microenvironment through RNA sequencing analysis. Considering the pathological changes caused by hypoxia in bone defects, we aimed to develop a construct for oxygen supply, incorporating ROS‐scavenging capabilities through utilizing an engineered “integrated multipurpose” hydrogel microsphere system（Scheme 1). Specifically, the composite nanoparticles were fabricated with a CaO_2_ core serving as an “oxygen reservoir”, and sequentially coated with SiO_2_ and PDA to construct a hierarchical structure resembling a nut chocolate. This design ensured a sufficient release of oxygen from the core, while the SiO_2_@PDA (SP) outer shell enabled a controllable hydrolysis reaction of CaO_2_, effectively converting hazardous intermediate substances and excessive ROS into oxygen. Subsequently, the CaO_2_@SiO_2_@PDA (CSP) nanoparticles were efficiently integrated with porous GelMA microspheres via amide bonds, thereby conferring continuous slow‐release oxygen properties and favorable ROS scavenging abilities. This approach could enhance the effectiveness of functionalized microspheres (CSP‐GM) in promoting cellular survival, stimulating osteoblast and endothelial cell activation, and alleviating oxidative stress within the bone defect microenvironment. Furthermore, the potential mechanism underlying antioxidant stress was explored. Finally, the bone regenerative capacity of nanocomposite microspheres was demonstrated using a rat femoral condyles bone defect model. Benefiting from its stable oxygen release and potent ROS scavenging ability, the multifunctional microspheres system exhibited promising potential in enhancing cell survival, restoring mitochondrial function, and attenuating oxidative stress under hypoxic conditions. More importantly, the system demonstrated a favorable capability to enhance angiogenesis and osteogenesis, indicating that the combined effects of oxygen supply and ROS‐scavenging could break the vicious cycle of hypoxia‐oxidative stress to remodel the regeneration microenvironment, thus playing a synergistic therapeutic role in the treatment of bone defects. In summary, our findings suggested that this novel micro‐oxygen reservoir system, integrating oxygenating nanomaterials within biocompatible hydrogel microspheres, presented a promising strategy for bone tissue regeneration.

## 2 Results and Discussion

### 2.1 Transcriptome Sequencing for Investigating the Potential Mechanisms of Hypoxia Induced Bone Metabolism

**Figure 1:**
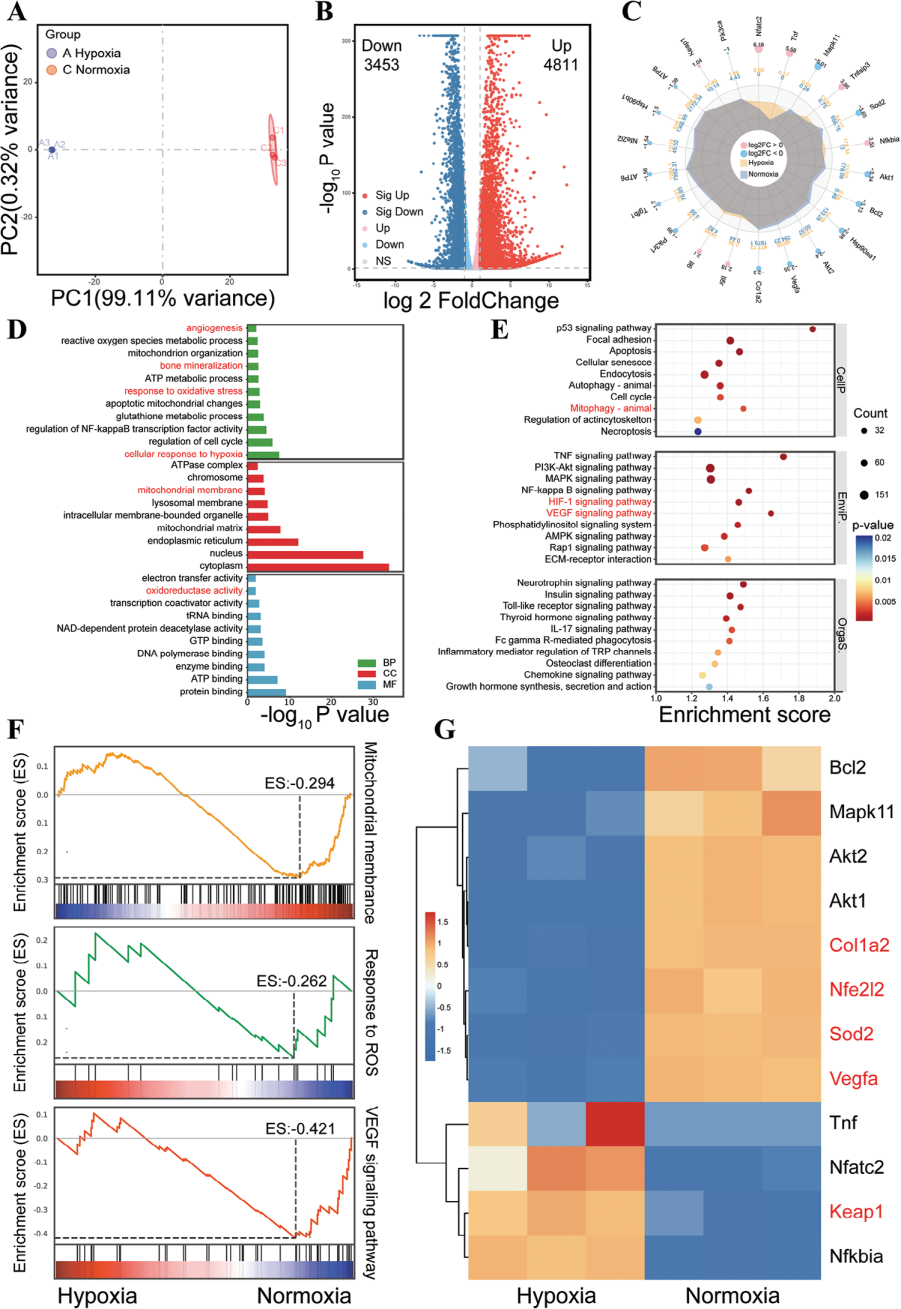
Transcriptome sequencing analysis of BMSCs cultured in hypoxia versus normoxia microenvironments. A) Volcano map of DEGs. B) Radar plot of differential gene expression levels. C) GO enrichment analysis of DEGs. D) KEGG enrichment analysis of DEGs. E) Selected GSEA analysis. F) Heat map of DEGs.

Precise regulation of microenvironmental pathophysiologic process in bone defects is crucial for effective bone repair. To explore the impact of hypoxia on potential cellular mechanisms and associated biological events, we conducted RNA sequencing to explore the difference of BMSCs gene expression upon hypoxia and normoxia conditions. The principal component analysis (PCA) showed good reproducibility among samples from each group (Figure S1A, Supporting Information). The volcano plot of differentially expressed genes (DEGs) exhibited a total of 3453 downregulated DEGs and 4811 upregulated DEGs in the hypoxia group compared with the normoxia group (Figure 1A). Furthermore, radar plot (Figure 1B) and heatmap analysis (Figure S1B, Supporting Information) revealed significant differences in gene expression between the two groups, indicating that hypoxia significantly impacted gene expression profile of BMSCs. The DEGs were subjected to Gene Ontology (GO) analysis, which encompassing the classifications of biological process (BP), cellular component (CC), and molecular function (MF). Enrichment analysis revealed dysregulation in various biological functions, such as angiogenesis, bone mineralization, mitochondrial membrane, and response to oxidative stress, reflecting the multifaceted detrimental effects on bone regeneration impaired by hypoxia (Figure 1C; Figure S2, Supporting Information).

Kyoto Encyclopedia of Genes and Genomes (KEGG) enrichment analysis was utilized to investigate the potential signaling pathways underlying these functional changes. The signaling pathways associated with cell function and proliferation (e.g., mitophagy, apoptosis and the p53 signaling pathway), oxidative stress (e.g., PI3K‐Akt signaling pathway^[^ ^23^ ^]^), and angiogenesis (involving HIF‐1 and VEGF signaling pathway) were found to be significantly impacted (Figure 1D). Gene set enrichment analysis (GSEA) was further performed. Consistent with the aforementioned findings, down‐regulation of mitochondrial membrane, response to ROS, and VEGF signaling pathway (Figure 1E), and up‐regulation of response to hypoxia were observed in the hypoxia group (Figure S3, Supporting Information). Furthermore, a heat map displayed that representative DEGs in these signaling pathways were either down‐regulated or up‐regulated correspondingly (Figure 1F). The transcriptome sequencing results demonstrated that hypoxia affected proliferation and mitochondrial function of BMSCs, while concurrently inhibiting antioxidant and angiogenic activity, ultimately compromising osteogenesis. Therefore, biomaterials engineered to mitigate hypoxia and oxidative stress exhibit significant potential in promoting bone regeneration.

### 2.2 Synthesis and Characterization of CSP Nanoparticles with ROS Scavenging Capacity

**Figure 2:**
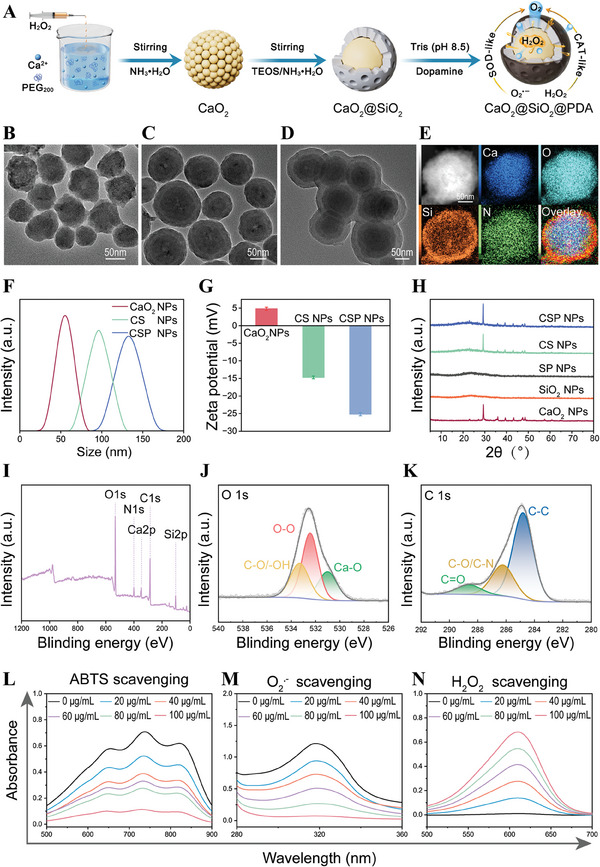
Synthesis and characterization of the composite oxygenating nanoparticles. A) Structural diagram of the synthesis of CSP. Representative TEM images of B) CaO_2,_ C) CS and D) CSP, respectively. E) Corresponding EDS elemental mapping of CSP. F) Size distribution and G) Zeta potentials of CaO_2_, CS and CSP (n = 3). H) XRD patterns of CaO_2_, SiO_2,_ SP, CS and CSP. XPS spectra of I) CSP, J) O 1S, and K) C 1S of CSP. Scavenging of L) ABTS, M) O_2_^•−^, and, N) H_2_O_2_ with different concentration of CSP nanoparticles.

Following the synthesis process depicted in Figure 2A, the CSP nanoparticles (NPs) were synthesized and characterized using Transmission electron microscopy (TEM) and Energy‐dispersive X‐ray spectroscopy (EDS) elemental mapping. The TEM images confirmed the distinct core‐shell structure of the CSP NPs, illustrating the progressive encapsulation of the CaO_2_ core with SiO_2_ and PDA layers (Figure 2B–D). EDS mapping elucidated the spatial distribution of calcium (Ca), oxygen (O), silicon (Si), and nitrogen (N) across the nanoparticles, with the merged image further confirming the successful synthesis of CSP NPs (Figure 2E). Dynamic Light Scattering (DLS) measurements (Figure 2F) delineated the incremental growth in hydrodynamic diameter from the pristine CaO_2_ (64.33±1.39 nm) through the SiO_2_‐coated stage (112.80±2.13 nm) to the final CSP NPs (150.27±2.12 nm), consistent with TEM findings. Additionally, the TEM images and DLS confirmed the successful synthesis of SP nanoparticles (Figures S4 and S5, Supporting Information). Moreover, the changes in zeta potential indicated the successful amination modification and shell coating processes throughout the synthesis stages. As shown in Figure 2G, the zeta potential of raw CaO_2_ nanoparticles was measured to be 4.81±0.45 mV, which shifted to ‐14.70±0.40 mV post‐SiO_2_ coating, and further to ‐25.17±0.42 mV upon PDA coating. The SiO_2_ shell was modified with amino groups to enhance hydrophilicity and facilitate the attachment of the CaO_2_@SiO_2_ (CS) construct onto GelMA hydrogel, as evidenced by the shift of zeta potential from negative to positive values (Figure 2G; Figure S6, Supporting Information). Supplementary validation of the amino group modification on the CS NPs was furnished by Fourier transform infrared (FTIR) spectroscopy. As shown in Figure S7 (Supporting Information), the emergence of N‐H vibrations peaks at 1636 cm^−1^ attributed to the stretching modes of the NH_2_ groups, confirmed functionalization of the SiO_2_ shell with amino groups.

To substantiate the elemental composition of the CSP NPs, X‐ray diffraction (XRD) and X‐ray photoelectron spectroscopy (XPS) analyses were conducted. The XRD pattern (Figure 2H) presented characteristic diffraction peaks at 2θ values of 29.8°, 36.2°, 48.0°, and 61.5°, corresponding closely to the (002), (110), (112), and (202) facets of CaO_2_ (JCPDS PDF card NO. 03–0865). Conversely, the SP layer displayed an amorphous crystal structure. The deposition of SP layer onto CaO_2_ core did not change the peak position or introduce additional diffraction peaks, thereby confirming the successful loading of amorphous SP onto CaO_2_ while preserving its crystalline structures integrity. The XPS survey spectra (Figure 2I) elucidated the composition and chemical state of nanoparticles, highlighting the presence the presence of Ca, O, Si, C, and N, aligning with the EDS elemental mapping results. The Ca 2p spectrum exhibited two peaks at 347.4 eV and 350.9 eV corresponding to the Ca 2p3/2 and Ca 2p1/2, indicative of the divalent state of calcium (Ca^2+^) (Figure S8A, Supporting Information). Furthermore, O‐O was observed at 532.4 eV in high‐resolution spectrum of O 1s, indicating the presence of peroxide groups (Figure 2J).^[^ ^24^ ^]^ And the Si 2p peaks at 103.3 eV corresponded to Si‐O bonds within the SiO_2_ shell (Figure S8B, Supporting Information). As shown in Figure 2K, the peaks at 284.8, 286.2, and 288.6 eV in the C 1s spectrum corresponded to C–C, C‐O/C‐N, and C = O groups respectively in PDA coating. The C = O peak at 288.6 eV could be attributed to the oxidation of catechol during the self‐polymerization of PDA.^[^ ^25^ ^]^ The N 1s spectrum, with peaks at 398.7 and 400.0 eV attributed to C‐N and ‐NH_2_ species within the PDA layer, respectively (Figure S8C, Supporting Information). Collectively, these findings validated the successful synthesis and coating of the CaO_2_ NPs with SiO_2_ and PDA coating.

Impaired bone regeneration is closely associated with the excessive accumulation of ROS and the subsequent oxidative stress damage. Coating biomaterials with PDA is an effective strategy for endowing ROS scavenging capability. The potential antioxidant activity of CSP NPs was assessed through a series of free radical‐scavenging assays. The overall antioxidant activity was evaluated using 2,2′‐azinobis‐3‐ethylbenzothiazoline‐6‐sulphonate (ABTS) radical scavenging assay, and a concentration‐dependent decrease in absorbance at 734 nm indicated the potent free radical scavenging ability of CSP NPs (Figure 2L). Additionally, the ability of CSP nanoparticles to neutralize superoxide anions (O_2_^•−^) was analyzed, revealing a notable reduction in the absorption peak at 320 nm, which demonstrated their effectiveness in O_2_^•‐^ depletion. Consistent with the ABTS assay outcomes, the O_2_^•−^ scavenging activity was found to be concentration‐dependent (Figure 2M). Furthermore, the H_2_O_2_ scavenging capacity of the CSP NPs was evaluated using indigo carmine as an indicator. In the absence of CSP NPs, there was a sharp decline in the absorption peak at 610 nm due to dye decomposition in the presence of H_2_O_2_. However, the introduction of CSP NPs resulted in a significant preservation of absorbance at 610 nm, highlighting their remarkable H_2_O_2_ scavenging efficiency (Figure 2N). These results demonstrated the superior radical scavenging capability of CSP nanoparticles, positioning them as promising antioxidant agents for effectively mitigating harsh oxidative stress in bone defects.

### 2.3 Preparation and Characterization of Composite Oxygenating Hydrogel Microspheres

**Figure 3:**
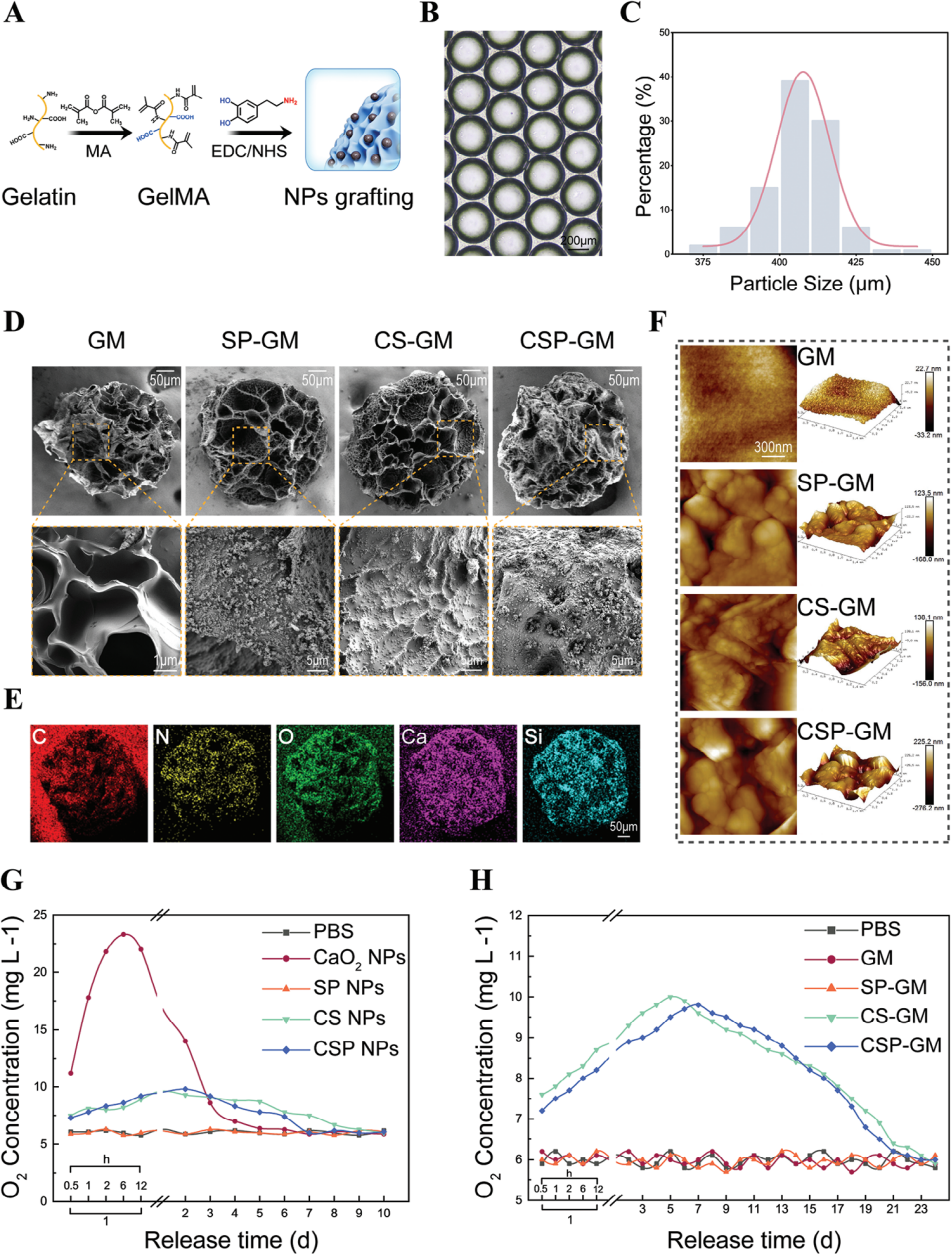
Preparation and characterization of composite microspheres. A) Schematic for the process of grafting nanoparticles to GelMA microspheres. B) Morphology of GelMA microspheres under light microscope. C) Particle size distribution of GelMA microspheres (n = 100). D) SEM images and magnified surface morphology of different microspheres. E) Elemental mapping images of CSP‐GM. F) The surface roughness of different microspheres scanned by AFM. Oxygen release curves of different G) nanoparticles and H) microspheres in PBS for different times (n = 3).

GelMA hydrogel has been widely utilized in the fields of tissue engineering and regenerative medicine due to its exceptional biocompatibility and degradability. Furthermore, GelMA microspheres with controllable particle size and abundant porous structure can be engineered using microfluidic technology and lyophilization.^[^ ^21b^ ^]^ In our study, GelMA microspheres, as ideal carriers, which provided a good basis for nanoparticles grafting to effectively enhance their functionality and achieve sustained release capability. Within a 1‐ethyl‐3‐[3‐dimethylaminopropyl] carbodiimide hydrochloride (EDC)/N‐hydroxy succinimide (NHS) coupling system, the amino groups existing in PDA were cross‐linked with the carboxyl group of GelMA microspheres, facilitating the successful grafting of CSP NPs and the fabrication of multifunctional micro‐nano microspheres (CSP‐GM, Figure 3A). The light microscopy images confirmed the uniform dispersion and consistent morphology of the hydrogel microspheres, with an average particle size were 406.26±11.28 µm (Figure 3B,C). The lyophilized hydrogel microspheres were further examined using scanning electron microscopy (SEM) and EDS. As shown in Figure 3D, the microspheres exhibited regular morphology and microporous structure, indicating their potential as a conducive living niche for cell growth and proliferation. Local magnification of the microspheres revealed a visibly rough surface and the uniform attachment of nanoparticles, suggesting effective loading following biomimetic surface grafting. EDS analysis clearly revealed uniform distribution of corresponding C, N, O, Ca, and Si elements in CSP‐GelMA microspheres (Figure 3E), contrasting with the simpler elemental profile of C, N, and O elements in the unmodified GelMA microspheres (Figure S9, Supporting Information). Subsequently, atomic force microscope (AFM) was employed to evaluate the surface roughness and nanoscale topology of the composite microspheres. Consistent with SEM findings, AFM observation provided similar information on surface structure (Figure 3F). Protrusions resulting from nanoparticles incorporation were observed in the composite microspheres with higher bulging degrees due to the presence of nanoparticles. Therefore, our findings had demonstrated the successful grafting of nanoparticles onto GelMA microspheres. Furthermore, in the in vitro degradation experiments, after 4 weeks of incubation, the microspheres exhibited a time‐dependent degradation profile. In the presence of SiO_2_@PDA coating from nanoparticles, the degradation rate of the GelMA microspheres was slightly decreased, which might be attributed to the physical barrier effect exerted by the nanoparticles grafted onto the microspheres surface (Figure S10, Supporting Information).^[^ ^21d^ ^]^ As the microspheres degraded gradually in a surface corrosion mode, progressing from the outer surface to the core. The results revealed the degradation performance of the composite microspheres, suggesting their potential as an effective sustained‐release system.

### 2.4 In Vitro Oxygen Release Behaviors of Oxygenating System

The burst release of oxygen can lead to adverse effects due to excessively high local concentrations, while insufficient protection may cause a rapid depletion of oxygen supply within biomaterials. As shown in Figure 3G, the O_2_ release from CaO_2_ nanoparticles initially surged, reaching a peak at 23.3 mg L^−1^ within the first 12 h, and then experienced an obvious decline, returning to baseline levels after 3 days under physiological conditions (pH 7.4, 37 °C). The encapsulation within a hydrophobic shell significantly enhanced the stability and duration of the oxygen release, extending it to nearly a week, which suggested that the silica shell effectively modulated and prolonged the release of oxygen. More importantly, when integrated with GelMA microspheres, a significantly prolonged and sustained release of O_2_ over 20 days was observed (Figure 3H). This extended‐release profile is advantageous for maintaining cell viability and fostering a conducive environment for angiogenesis and osteogenesis under hypoxic conditions.

### 2.5 Protection of BMSCs by Composite Oxygenating Hydrogel Microspheres under Hypoxia Microenvironment

**Figure 4:**
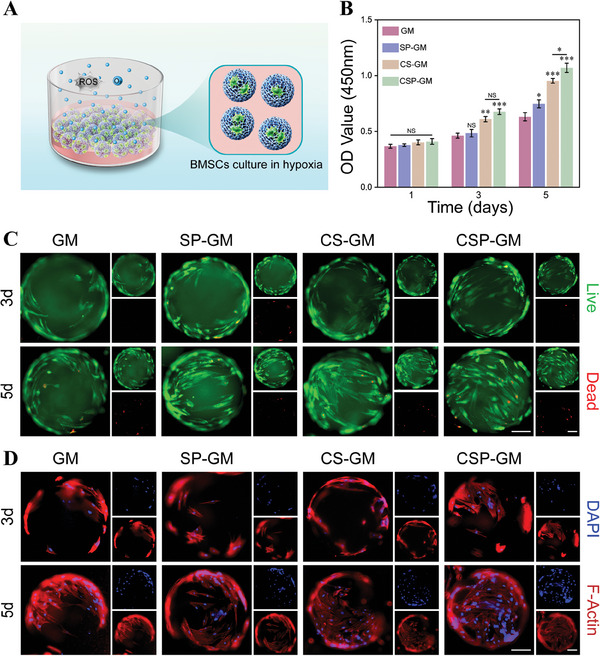
The biocompatibility and cell protection of composite microspheres in hypoxia microenvironment. A) Schematic diagram of the proliferation and adhesion effects of microspheres on BMSCs in hypoxia microenvironment. B) The proliferation of BMSCs on 1,3, and 5 days detected by CCK‐8 assay (n = 3). C) Live/Dead (scale bar, 100 µm) and D) Cytoskeleton (F‐actin, scale bar, 100 µm) fluorescence staining of BMSCs cultured on microspheres for 3 and 5 days. NS: no significance, ^*^ *p* < 0.05, ^**^ *p* < 0.01, and ^***^ *p* < 0.001 compared with the GM group.

The oxygen tension within the fracture haematoma is notably lower than in peripheral blood, which decreases steeply during the first week post‐fracture.^[^ ^2b^ ^]^ In the damaged bone, hypoxia severely impedes the survival and differentiation of BMSCs, ultimately impairing bone regeneration. To evaluate the protective effect of oxygenating microspheres on BMSCs in hypoxia environment, the cells were co‐cultured with different types of microspheres within an Anaeropack anaerobic chamber. This system enabled an oxygen concentration of less than 1% while maintaining carbon dioxide levels at ≈5% (Figure 4A).^[^ ^4b^ ^]^ The CCK‐8 assay results (Figure 4B) indicated that BMSCs co‐cultured with microspheres exhibited comparable proliferative activity on the first day across all groups. After 5 days of co‐incubation, the cell proliferation rates in the groups treated with composite microspheres were significantly higher than that in the GM (GelMA microsphere) group. Among these, the CSP‐GM group exhibited the most pronounced increase in cell proliferation, followed by CS‐GM (CS‐GelMA microsphere) and SP‐GM (SP‐GelMA microsphere) groups.

Live/dead staining assays revealed a substantial increase in the viable cell count on the CSM‐GM surface as the incubation period extended to 3 and 5 days, while only minimal variation was observed in the density of living cells in the GM group (Figure 4C). It is noteworthy that a minimal number of dead cells were observed on the microspheres, likely because they were prone to detach from the surface upon losing integrity. Additionally, cytoskeletal staining highlighted the formation of F‐actin filaments and networks within live BMSCs that adhered to the hydrogel microspheres (Figure 4D). BMSCs cultured on pure GM surface exhibited restricted spreading, suggesting a compromised cell adhesion capability. Conversely, BMSCs that adhered to the CSP‐GM displayed improved spreading morphology, characterized by orderly cytoskeletal structures and a progressive increase in DAPI fluorescence intensity, indicating a significant increase in cell numbers. Stem cell viability was significantly inhibited under hypoxic conditions; however, it was preserved in the presence of composite microspheres, which possessed dual functions of oxygen generation and ROS scavenging capabilities. Consequently, the functionalized microspheres had the potential to mitigate the detrimental effects of hypoxia on cell survival and provide a favorable platform for in situ cell attachment and proliferation at the bone defect sites following implantation.

### 2.6 Antioxidative Stress Properties of Composite Oxygenating Hydrogel Microspheres under Hypoxia Microenvironment

**Figure 5:**
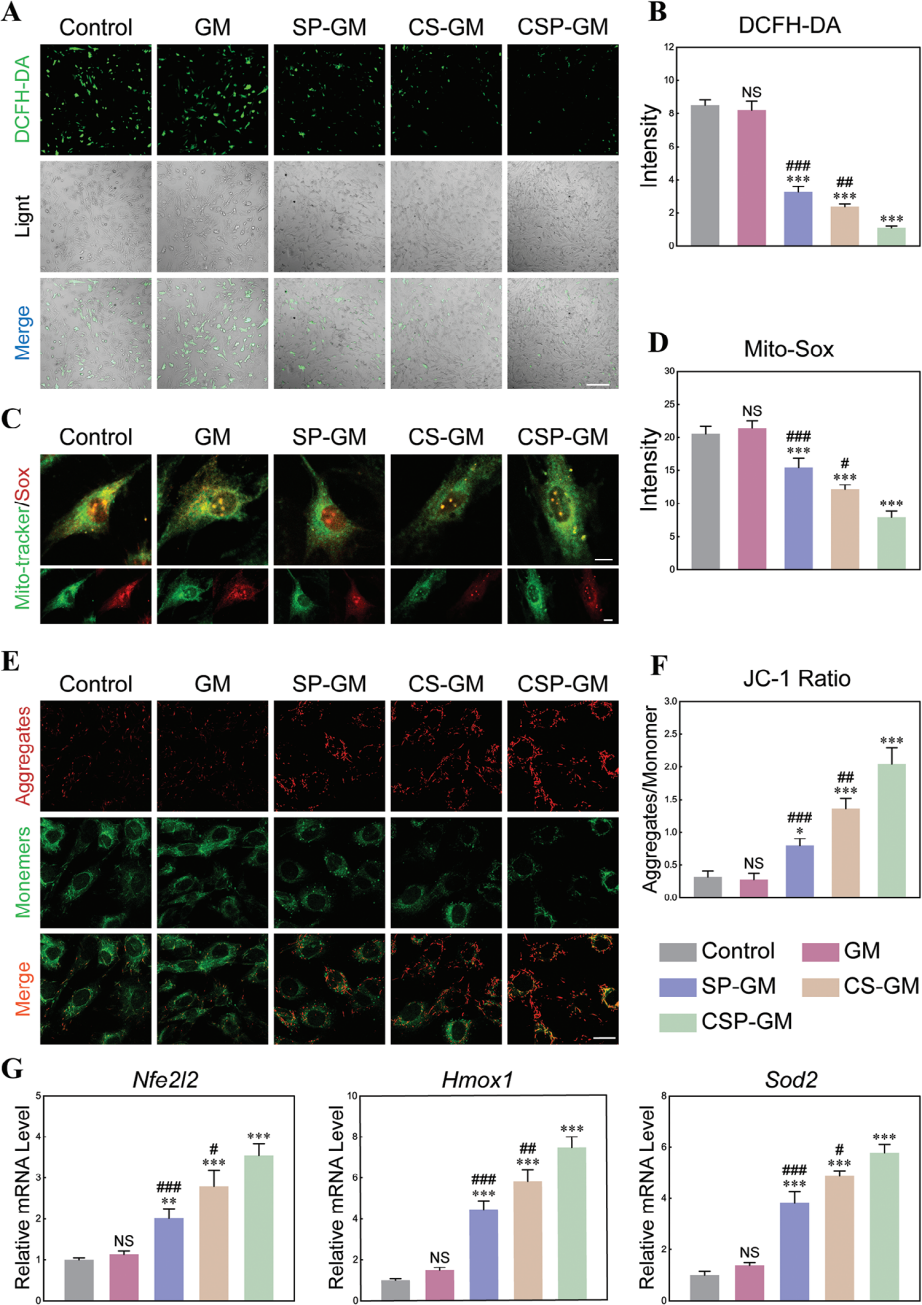
Intracellular ROS‐scavenging and antioxidant properties of composite microspheres in hypoxia microenvironment. A) Intracellular ROS fluorescence staining of BMSCs detected by DCFH‐DA probes (scale bar, 200 µm). B) Quantitative analysis of DCFH‐DA fluorescence intensity of BMSCs (n = 3). C) Mitochondrial ROS (MitoTracker/MitoSox) fluorescence staining (scale bar, 10 µm). D) Quantitative analysis of MitoSox fluorescence intensity (n = 3). E) Detection (scale bar, 20 µm) and F) quantitative analysis of JC‐1 Mitochondrial membrane potentials of BMSCs (n = 3). G) Relative mRNA expression of antioxidant‐related genes in BMSCs at 24 h detected by qRT‐PCR (*Nr2l2*, *Hmox‐1*, *Sod2*, n = 3). NS: no significance, ^*^ *p* < 0.05, ^**^ *p* < 0.01, and ^***^ *p* < 0.001 compared with the Control group; ^#^ *p* < 0.05, ^##^ *p* < 0.01, and ^###^ *p* < 0.001 compared with the CSP‐GM group.

The prolonged ischemia‐hypoxia microenvironment following bone injury triggers substantial ROS generation at bone defects site, potentially disrupting the intracellular antioxidant equilibrium and leading to profound oxidative stress. Consequently, the abnormal accumulation of ROS further exacerbates intracellular hypoxia and disordered microenvironment, creating a vicious cycle of hypoxia and oxidative stress that ultimately induces damage and apoptosis of reparative cells. To assess the intracellular ROS scavenging capacity of proposed microspheres, we utilized the ROS‐sensitive probe 2,7‐dichlorodihydrofluorescein diacetate (DCFH‐DA) to monitor the ROS level within BMSCs co‐cultured with microspheres in hypoxia environment. As shown in Figure 5A, significant decreases in fluorescence intensity were observed in the SP‐GM and CS‐GM groups compared to both positive control and GM groups, suggesting a lower level of intracellular ROS. Furthermore, BMSCs treated with CSP‐GM exhibited minimal green fluorescence, demonstrating the strongest ROS scavenging effect, which was further confirmed by quantitative analysis of DCFH‐DA fluorescence intensity (Figure 5B) and flow cytometry (Figure S11, Supporting Information). Furthermore, the excessive ROS production can induce lipid peroxidation, eliciting alterations in mitochondrial membrane potential (MMP) and mitochondrial dysfunction.^[^ ^18^, ^26^ ^]^ Since mitochondria are the primary sources of ROS within cells, we utilized MitoSox probes to evaluate mitochondrial ROS production in BMSCs. Concurrently, Mito‐Tracker Green probes were employed for mitochondrial staining. An increase in mitochondrial ROS, induced by hypoxia, was observed in both the control and GM groups. Leveraging their dual‐regulation effects on microenvironment, the multifunctional microspheres system demonstrated a superior capacity to mitigate both intracellular and mitochondrial oxidative stress in BMSCs. The mitochondrial ROS levels were significantly reduced in BMSCs co‐cultured with composite microspheres, with the CSP‐GM group showing the most pronounced decrease (Figure 5C,D).

The GO and KEGG analyses revealed that hypoxia profoundly affected mitochondrial function, resulting in changes in mitochondrial membrane and mitophagy progress. Furthermore, GSEA analysis indicated an up‐regulation effect on mitochondrial membrane of BMSCs treated with CSP‐GM under hypoxia conditions (Figure S13A, Supporting Information). Subsequently, the JC‐1 assay was performed to determine the MMP of BMSCs. Transition from JC‐1 aggregates (red fluorescence) to JC‐1 monomers (green fluorescence) signifies the depolarization of mitochondrial transmembrane potential and a decline in MMP. A higher presence of JC‐1 monomers was observed in the control group, while BMSCs co‐cultured with the composite microspheres exhibited an increase presence of JC‐1 monomers coupled with a reduced presence of JC‐1 aggregates (Figure 5E). Additionally, the quantitative analysis (Figure 5F) demonstrated that CSP‐GM significantly elevated MMP levels when compared to the SP‐GM and CS‐GM groups. This suggested the synergistic effect of ROS regulation and oxygen supply by CSP‐GM substantially improved mitochondrial function of hypoxic BMSCs.

Considering the capacity of composite microspheres to scavenge intracellular ROS and restore mitochondrial function, we further explored their underlying antioxidant mechanism. The heat map of DEGs showed that the expression levels of Kelch‐like ECH‐associated protein 1 (*Keap1*) were up‐regulated under hypoxia conditions, but significantly decreased after treatment with CSP‐GM (Figure S13B, Supporting Information). Acting as an oxidative stress sensor, the reduction of Keap1 indicates a decrease in oxidative stress.^[^ ^27^ ^]^ Furthermore, the expression levels of nuclear factor erythroid 2‐related factor 2 (*Nfe2l2*) and the antioxidant gene superoxide dismutase 2 (*Sod2*) were found to be upregulated after treatment with CSP‐GM compared with the control group (Figure S13B, Supporting Information). This implied that the anti‐oxidative stress effect of CSP‐GM may be attributed to Keap1 depletion activation and Nfe2l2 augmentation. Nfe2l2, a principal transcription factor regulating antioxidant stress, which can be activated to increase the expression of multiple antioxidant enzyme genes, including *Sod2* and heme oxygenase 1 (*Hmox1*) after dissociation from Keap1.^[^ ^27^ ^]^ Previous studies have demonstrated that Nrf2/HO‐1 signaling pathway axis play an important role in cellular response to oxidative stress.^[^ ^28^ ^]^ Conversely, heightened mtROS levels can inactivate the Nrf2‐activating signaling cascade, thereby inhibiting Nrf2 activity.^[^ ^29^ ^]^ To determine whether the composite microspheres exerted antioxidant effects via Nrf2/HO‐1 signaling pathway, we proceeded to evaluate the expression levels of *Nfe2l2* and its downstream genes by qRT‐PCR. As shown in Figure 5G, the expression levels of *Nfe2l2, Sod2*, and *Hmox1* in BMSCs treated with composite microspheres significantly increased compared to the control and GM groups, suggesting that the functionalized microspheres may enhance resistance to oxidative stress in BMSCs by upregulating the expression of antioxidant genes. Furthermore, the expression levels of Nrf2 and its downstream proteins were evaluated by western blotting assay. These results were consistent with those from RNA sequencing and qRT‐PCR analysis. The expression of Nrf2 protein and antioxidant proteins HO‐1 and SOD2 was significantly inhibited in the control and GM groups. However, treatment with composite microspheres significantly reversed the inhibitory effects, with the CSP‐GM group exhibiting the highest protein expression levels among all the groups (Figure S14, Supporting Information). Our results demonstrated that, besides multi‐tiered neutralization of excessive ROS, CSP‐GM possessed the potential to enhance intrinsic antioxidant capacity of BMSCs via the Keap1/Nrf2 signaling pathway. Taken together, we had elucidated the underlying mechanisms responsible for the functional recovery of BMSCs under hypoxia conditions. It had been demonstrated that CSP‐GM fostered a conductive microenvironment for BMSCs by breaking the vicious cycle involving ROS overgeneration, mitochondrial dysfunction and the degradation of antioxidant defenses.

### 2.7 Angiogenesis Effect of Composite Oxygenating Hydrogel Microspheres under Hypoxia Microenvironment

**Figure 6:**
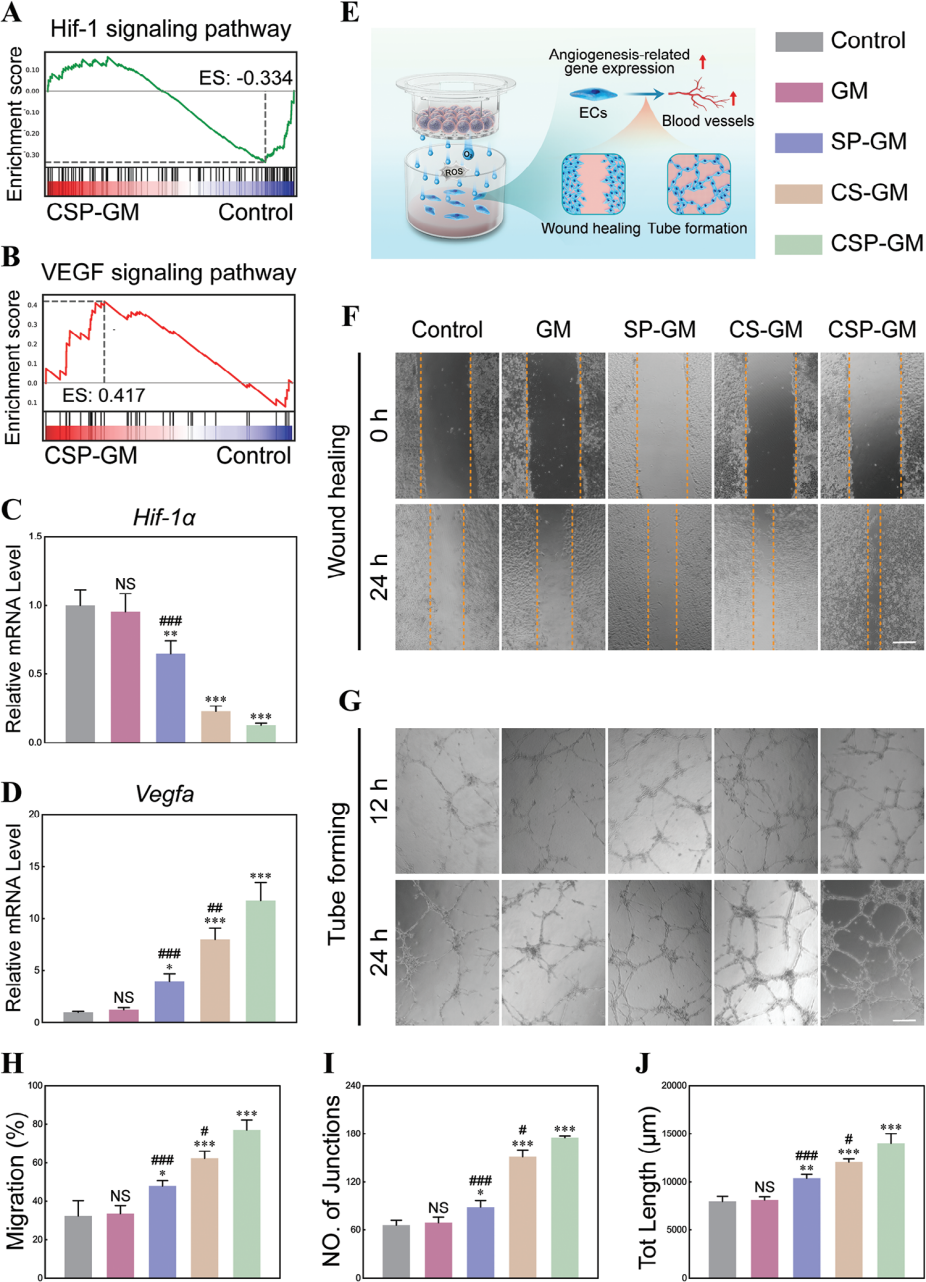
Angiogenic activities of composite microspheres in hypoxia microenvironment. GSEA analysis of A) Hif‐1 and B) VEGF signaling pathway in BMSCs. Relative mRNA expression of C) *Hif‐1α* and D) *Vegfa* in BMSCs at 24 h detected by qRT‐PCR (n = 3). E) Schematic diagram of the angiogenesis of microspheres on HUVECs in hypoxia microenvironment. F) Evaluation of HUVECs migration by wound healing assay (scale bar, 500 µm). G) Matrigel tube formation assays of HUVECs (scale bar, 500 µm). H) Quantitative analysis of wound healing assay (n = 3). Quantitative analysis of tube formation, including I) Number of junctions, and J) Total length (n = 3). NS: no significance, ^*^ *p* < 0.05, ^**^ *p* < 0.01, and ^***^ *p* < 0.001 compared with the Control group; ^#^ *p* < 0.05, ^##^ *p* < 0.01, and ^###^ *p* < 0.001 compared with the CSP‐GM group.

Revascularization plays a crucial role in bone healing, as it ensures the delivery of nutrients to the injured site and protects functional cells from damage caused by hypoxia and associated aberrant ROS production, influencing bone regeneration.^[^ ^4^, ^7^ ^]^ Furthermore, inadequate vascularization poses a significant barrier to the optimal performance of tissue engineering implants.^[^ ^30^ ^]^ Therefore, well‐designed functional biomaterials should exhibit favorable pro‐antigenic activities to promote bone healing. The relationship between hypoxia and angiogenesis was initially assessed through RNA sequencing. GSEA analysis revealed that CSP‐GM treatment up‐regulated the VEGF signaling pathway in BMSCs compared with those subjected to hypoxia conditions (Figure 6A,B). Moreover, the heat map exhibited that *Vegfa* gene expression was down‐regulated under hypoxic conditions, but was upregulated with CSP‐GM treatment (Figure S13B, Supporting Information). On this basis, the mRNA expression levels of *Hif‐1α* and *Vegfa* were further analyzed with qRT‐PCR assays. Hif‐1α, a key regulator of oxygen homeostasis, mediates the cellular response to fluctuations in oxygen levels. It is expected to be upregulated at the fracture site due to local hypoxia and blood supply deficiency following injury. Similar to previous findings, both control and GM groups displayed the higher levels of *Hif‐1𝛼* expression.^[^ ^11^, ^31^ ^]^ Composite microspheres with synergistic oxygen generation and ROS scavenging properties, effectively alleviated cell hypoxia, resulting in an 87.42% reduction in *Hif‐1𝛼* expression levels (Figure 6C). Generally, Hif‐1α can stimulate Vegf expression,^[^ ^17^ ^]^ whereas prolonged hypoxia may result in decreased expression of Vegf.^[^ ^7^, ^31^ ^]^ The regulatory effect of Hif‐1α on cell proliferation under hypoxia depends is contingent upon the severity or duration of hypoxia, as it exhibits an inhibitory effect in prolonged hypoxia.^[^ ^32^ ^]^ Although the expression of *Hif‐1𝛼* was reduced in the groups treated with nanoparticle‐modified microspheres compared to the control and GM groups, a markedly elevated *Vegfa* expression was observed, with the CSP‐GM group exhibiting the highest expression level (Figure 6D). As Chen et al.’s study explored, prolonged hypoxia (>9 h) would result in disrupted spatiotemporal oxygen homeostasis and a decline in *Vegfa* gene expression.^[^ ^7b^ ^]^

Endothelial cells (ECs) are essential for vascularization, and the angiogenic potential of composite microspheres was further evaluated using human umbilical vein endothelial cells (HUVECs) through scratch wound healing and tube formation assays (Figure 6E). Obvious cell migration and wound closure were observed in groups treated with composite microspheres, except for the control and GM group at a 24‐hour interval (Figure 6F). The 24‐hour migration rate (Figure 6H) for the CSP‐GM group (77.05±5.07%) was significantly higher than that of the SP‐GM and CS‐GM groups (47.99±2.68% and 62.20±3.75%, respectively). These results implied that ECs motility was compromised in hypoxic environment, but the combined action of oxygen generation and ROS scavenging by CSP‐GM significantly promoted accelerated migration of ECs and gap closure. In addition, only incomplete tubes were observed in the control and GM group, indicating constrained vasculogenic activity of ECs (Figure 6G). Despite the absence of direct contact between the composite microspheres and ECs within the Transwell no‐contact coculture system, our results indicated that persistent O_2_ release significantly facilitated the tube formation process of ECs, as demonstrated by the development of interconnected vascular‐like network structures over time. Quantitative analysis of newly formed tube networks revealed significant enhancements across several metrics, including the number of junctions, total vessels length, and number of meshes across different treatment groups (Figure 6I,J; Figure S15A,B, Supporting Information). The impaired angiogenic capabilities could be mitigated by SP‐GM (for ROS scavenging) and CS‐GM (for oxygen supply), with CSP‐GM demonstrating the most pronounced proangiogenic effect among the tested groups. Overall, these findings highlighted the superior proangiogenic potential of biomaterials designed with a dual functionality of sustained oxygen release and ROS neutralization, effectively ameliorating the impaired angiogenesis in hypoxia status.

### 2.8 Osteogenesis Effect of Composite Oxygenating Hydrogel Microspheres under Hypoxia Microenvironment

**Figure 7:**
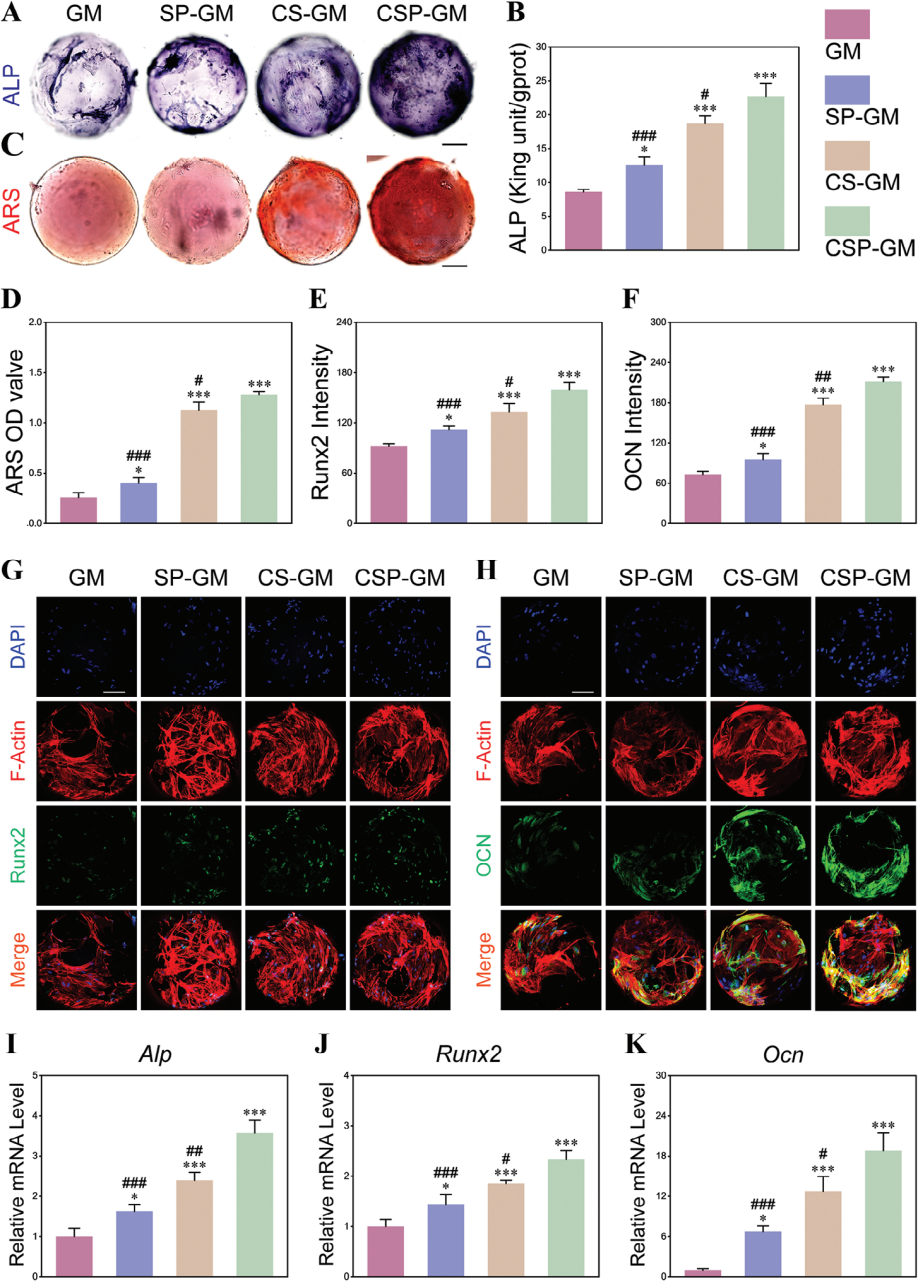
Osteogenic activities of composite microspheres in hypoxia microenvironment. A) ALP staining of individual microsphere 7 days after osteogenic induction (scale bar, 100 µm). B) Quantitative analysis of ALP activity (n = 3). C) ARS staining of individual microsphere 14 days after osteogenic induction (scale bar, 100 µm). D) Quantitative analysis of the calcium nodule content (n = 3). Quantitative analysis of E) Runx2 and F) OCN immunofluorescence staining intensity of microspheres (n = 3). G) Runx2 immunofluorescence staining of individual microsphere (scale bar, 100 µm). H) OCN immunofluorescence staining of individual microsphere (scale bar, 100 µm). Relative osteogenesis‐related mRNA expression of mRNA expression of I) *Alp*, J) *Runx2* at 7 days, and K) *Ocn* at 14 days detected by qRT‐PCR (n = 3). NS: no significance, ^*^ *p* < 0.05, ^**^ *p* < 0.01, and ^***^ *p* < 0.001 compared with the GM group; ^#^ *p* < 0.05, ^##^ *p* < 0.01, and ^###^ *p* < 0.001 compared with the CSP‐GM group.

Bone tissue engineered scaffolds designed with specific physical and biochemical cues, are instrumental in modulating cellular processes. Optimization of porous structure and surface topology, coupled with the sustained oxygen release and antioxidant properties, may enhance bone regeneration. To evaluate the effect of functionalized microspheres on osteogenic differentiation of BMSCs, we assessed the osteoinductive properties of these microspheres under hypoxic conditions in vitro at different time points. As an early osteogenic marker, alkaline phosphatase (ALP) activity was evaluated on day 7 after osteogenic induction. As shown in Figure 7A and S16A (Supporting Information), a significant increase of ALP expression was observed in the CS‐GM and CSP‐GM groups in contrast to the GM group. This trend was corroborated by a quantitative assessment of ALP activity, which revealed a significant and progressive enhancement across all experimental groups (Figure 7B). The capacity for bone mineral matrix formation, a crucial biological characteristic of advanced osteogenic differentiation, was evaluated using Alizarin Red S (ARS) staining to visualize calcium deposits after 14 days of co‐culture. The CSP‐GM group exhibited an abundance of calcium nodules, surpassing the CS‐GM and SP‐GM groups, with the GM group showing the sparsest calcium nodules (Figure 7C; Figure S16B, Supporting Information). Qualitative analysis revealed highest level of calcium deposition in CSP‐GM group, indicating its superior pro‐mineralization performance on BMSCs compared to the other groups (Figure 7D). To further assess the osteogenic capacity of cells within the microspheres, we employed immunofluorescence for runt‐related transcription factor 2 (Runx2), a key transcription factor involved in osteogenic differentiation, and osteocalcin (OCN), a key regulator of calcium metabolism. Furthermore, osteogenic potential of cells within the microspheres was evaluated through immunofluorescence of runt‐related transcription factor 2 (Runx2), a key transcription factor involved in osteogenic differentiation, and osteocalcin (OCN), a pivotal regulator of calcium metabolism. The immunofluorescence staining revealed a significant gradual increase in the fluorescence intensity of both proteins within the composite microspheres, particularly in the CSP‐GM group (Figure 7G,H). The expression levels of Runx2 and OCN in the CSP‐GM group were found to be ≈1.7‐fold and 2.9‐fold higher than those in the GM group respectively, with statistically significant difference (Figure 7E,F).

Moreover, the osteogenic differentiation potential of BMSCs was evaluated via qRT‐PCR analysis of osteogenesis‐related gene expression, including *Runx2*, *Alp*, collagen type‐I (*Col I*), and *Ocn*. As illustrated in Figure 7I–K and Figure S16C (Supporting Information), the expression level of *Runx2* in cells cultured on composite microspheres for 7 days was significantly increased, accompanied by elevated expression of two other early osteogenic genes *Alp* and *Col I*, as well as the late osteogenic marker gene *Ocn*. Importantly, a pronounced enhancement in the expression of these osteogenic genes was observed in the CSP‐GM group compared to the SP‐GM and CS‐GM groups, aligning with the findings from the ALP and ARS assessments. These compelling outcomes indicated that the synergistic effects of sustained oxygen generation and ROS scavenging significantly bolstered osteogenic differentiation at both the early and late stages within a hypoxia microenvironment.

Consequently, the functionalized microspheres system, with a unique hierarchical micro‐nano structure demonstrated commendable in vitro biological properties. On one hand, with biomimetic microarchitecture and excellent biocompatibility, it provided conducive structural support for the adhesion of repair cells and effectively served as a platform for signal transduction to regulate multiple cell functions including proliferation, differentiation and matrix deposition.^[^ ^33^ ^]^ On the other hand, the oxygen‐generating system integrated with ROS scavenging capabilities could remodel the compromised regenerative microenvironment, enhancing antioxidant activity and promoting angiogenesis and osteoblast differentiation under adverse hypoxia conditions.

### 2.9 In Vivo Antioxidative Stress Properties, Pro‐Angiogenesis and Pro‐Osteogenesis Capabilities of Composite Oxygenating Hydrogel Microspheres

**Figure 8:**
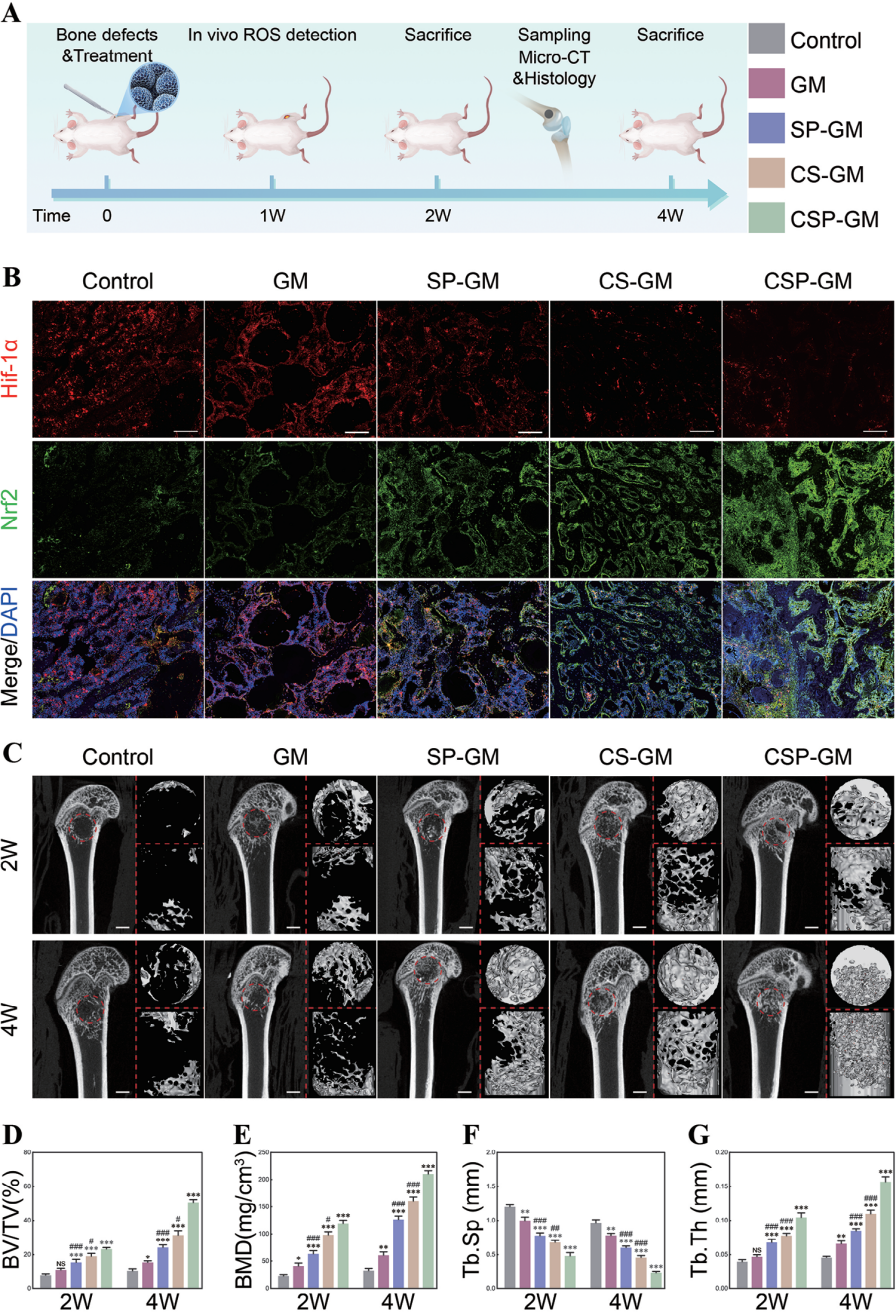
Bone regeneration of composite microspheres for critical femoral condylar defect in rats. A) Schematic diagram of composite microsphere implantation to critical femoral condylar defect in rats. B) Hif‐1α/Nrf2 immunofluorescence staining in defect area at 2 weeks postoperative (scale bars, 200 µm). C) 2D and 3D reconstruction images of femoral condyle defects at 2 and 4 weeks after implantation (scale bars, 1.5 mm). D‐G) Quantitative analysis of BV/TV, BMD, Tb.Sp, and Tb.Th scanned by Micro‐CT (n = 3). NS: no significance, ^*^ *p* < 0.05, ^**^ *p* < 0.01, and ^***^ *p* < 0.001 compared with the Control group; ^#^ *p* < 0.05, ^##^ *p* < 0.01, and ^###^ *p* < 0.001 compared with the CSP‐GM group.

In this study, we constructed a critical femoral condylar defect rat model to investigate the effect of microspheres on antioxidative stress and promotion of osteogenesis in vivo (Figure 8A). The hypoxia microenvironment at bone defect sites triggers oxidative stress, and reducing ROS level can alleviate oxidative stress and restore the homeostasis of endogenous antioxidant system. After one week of implantation, we initially used in‐vivo ROS imaging technique to assess the oxidative stress level in the defect area. As shown in Figure S17 (Supporting Information), the in‐vivo luminescence of bone defect area in the control and GM groups was much stronger compared to the groups treated with composite microspheres, while the lowest local ROS signal intensity was observed in the CSP‐GM group, indicating the best oxidative stress alleviating capability of the dual‐functional microspheres. Meanwhile, fluorescence staining of Hif‐1α demonstrated that, compared with the control group, the composite microspheres could significantly reduce the positive Hif‐1α expression levels in vivo (Figure 8B). Although both SP‐GM and CS‐GM were observed to partially alleviate the hypoxia at bone defects, there was still a significant disparity compared with the CSP‐GM group as evidenced by the quantitative analysis (Figure S18A, Supporting Information). Beyond reflecting antioxidant activity, the Nrf2 expression level is also associated with cellular hypoxia.^[^ ^4^, ^26^ ^]^ As shown in Figure 8B, the positive expression of Nrf2 was significantly upregulated in the groups treated with composite microspheres compared to the control and GM groups. Moreover, among the three composite microspheres groups, the CSP‐GM group exhibited the most pronounced increase in Nrf2 expression, which was corroborated by quantitative analysis (Figure S18B, Supporting Information). Sun et al.’s research reported similar findings, suggesting that Nrf2 expression was inhibited, while elevated hypoxia and ROS levels were observed at bone defect sites.^[^ ^4b^ ^]^ The variation of Nrf2 expression coincided with the levels of ROS and Hif‐1α, indicating that oxygenating microspheres with ROS‐scavenging properties could effectively enhance antioxidant activity and alleviate hypoxic conditions in vivo, laying a robust groundwork for subsequent tissue repair and regeneration.

The bone regeneration performance was evaluated using micro‐CT scanning at 2‐ and 4‐weeks following surgery. The sagittal views of femoral condyle (Figure 8C) revealed minimal new bone formation in the control group, with no radiographic signs of healing observed during the healing process. Conversely, defects treated with composite microspheres exhibited a greater amount of new bone ingrowth with satisfactory bone integration, and this difference significantly increased over time. The CSP‐GM group exhibited markedly superior repair outcomes at both time points, with the defect area entirely filled with new bone at 4 weeks, while visible deficiency was still present in the central area of the defects in the SP‐GM and CS‐GM groups. Micro‐CT 3D reconstructions provided a more precise visualization of new bone formation across the groups. The SP‐GM and CS‐GM groups exhibited better osteo‐inductive properties than GM group, with the greatest amount of new bone observed in the CSP‐GM group. These results suggested that the combination of oxygen release and ROS scavenging likely exerted synergistic effects on enhancing bone regeneration. Moreover, the analysis of bone volume/tissue volume (BV/TV) and bone mineral density (BMD) confirmed an increase in new bone formation within the defect area over time (Figure 8D–G). At 4 weeks, the BV/TV and BMD of CSP‐GM group were significantly higher than those of all other groups, indicating a larger volume of mature new bone formation in the defect area. Furthermore, an analysis of the trabecular bone microstructure parameters, including trabecular separation/spacing (Tb.Sp) and trabecular thickness (Tb.Th), was performed. As shown in Figure 8F,G, it was found that GM group exhibited higher Tb.Sp but lower Tb.Th values compared with SP‐GM and CS‐GM groups. Additionally, the CSP‐GM group demonstrated maximal Tb.Th and minimal Tb.Sp values, aligning with the morphological outcomes. Notably, radiographic results of GM group exhibited better bone regeneration compared to the control group, primarily due to the biomimetic microstructure of GelMA microspheres that enhanced osteoconductivity over the blank control group. Collectively, these results suggested that by multifaceted modulation of unbalanced microenvironment and excellent osteoconductivity, our proposed oxygen‐releasing microspheres system effectively promoted bone repair within defects, achieving optimal mineral density and structural integrity of newly formed bones.

**Figure 9:**
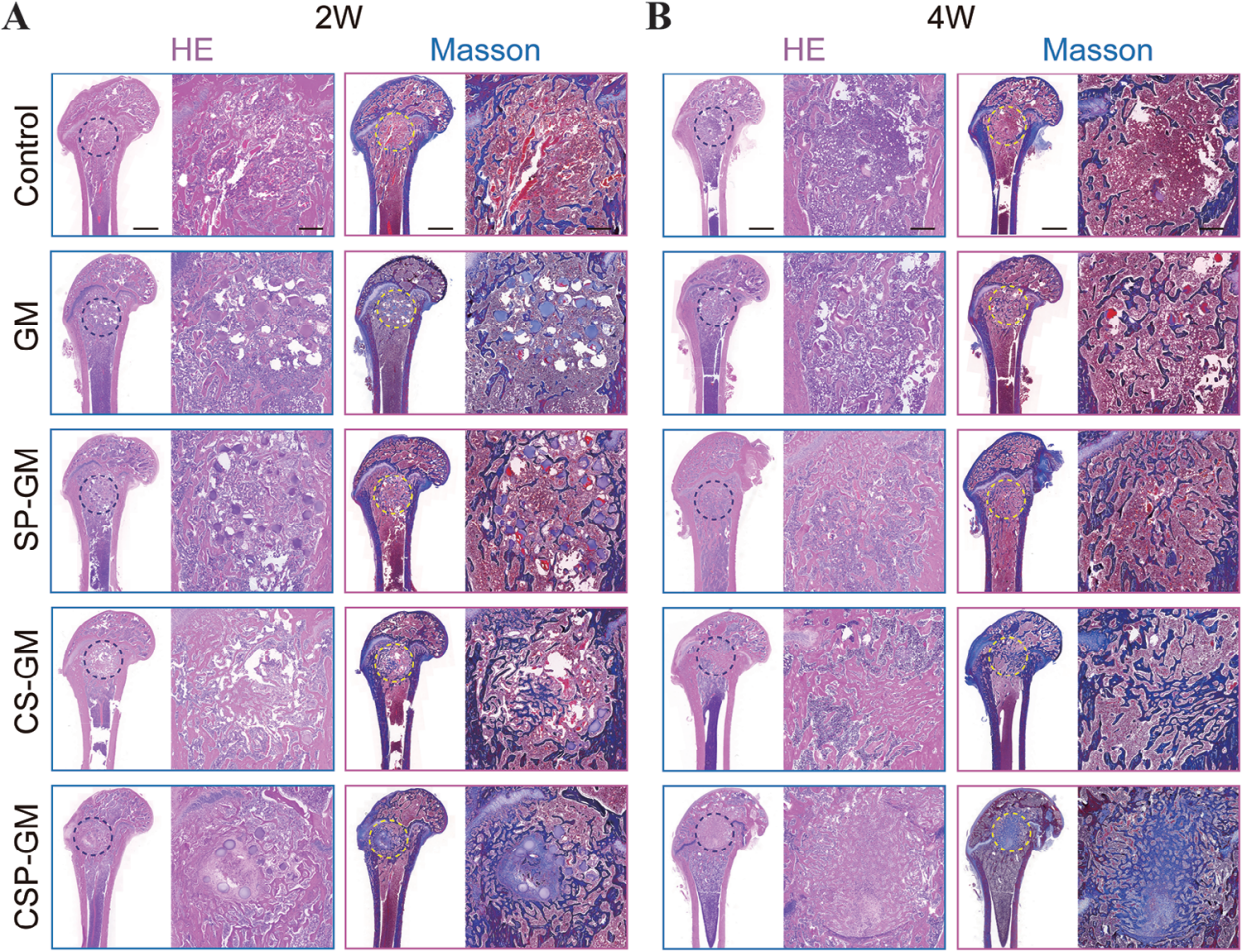
Histological staining of femoral condyle in rats for bone regeneration. Gross and magnified view of H&E staining and Masson staining of femoral condyle A) at 2 weeks postoperative. B) at 4 weeks postoperative. (Scale bar, 2 mm and 500 µm for gross and magnified images, respectively).

Hematoxylin & Eosin (H&E) and Masson's trichrome staining were employed to verify the histopathological structures of the regenerated new bone within the defect sites. In Figure 9A,B, sagittal sections of the femur and partially magnified images at 2 and 4 weeks were presented, respectively. As shown in H&E staining, the control group exhibited predominantly fibrous tissue formation in the defects at 2 weeks and only a limited amount of new bone tissue with scattered and discontinuous structures at the defect periphery after 4 weeks. Conversely, progressive regeneration of bone tissue was observed in the groups treated with microspheres over time. Notably, the CSP‐GM group showed a dense and orderly trabecular distribution within the defects, indicating significantly enhanced osteogenesis with a greater amount of bone matrix deposition at 4 weeks. Masson staining further revealed sparse deposition of blue‐stained collagen fibers in the original defects in the control group, while evident collagen fibers were observed in microspheres groups, suggesting localized new bone formation. As shown in Masson's staining, abundant and regularly arranged collagen deposition with continuous structures was observed in the CSP‐GM group, signifying the maturation and remodeling of the regenerated bone. The CS‐GM and SP‐GM groups showed similar but less pronounced effects. By 4 weeks after implantation, the residual hydrogel microspheres gradually degraded, being enveloped by newly formed bone tissue due to their ECM‐mimicking structures that offered a supportive framework for the ingrowth of new bone. Furthermore, the synergistic effects of oxygen delivery and ROS scavenging contributed to creating a conducive regenerative microenvironment that effectively regulated cellular activities and promoted bone regeneration. This discovery indicated that the functionalized microspheres could effectively facilitate repair cells ingrowth and enhance the biomaterial‐bone integration.

**Figure 10:**
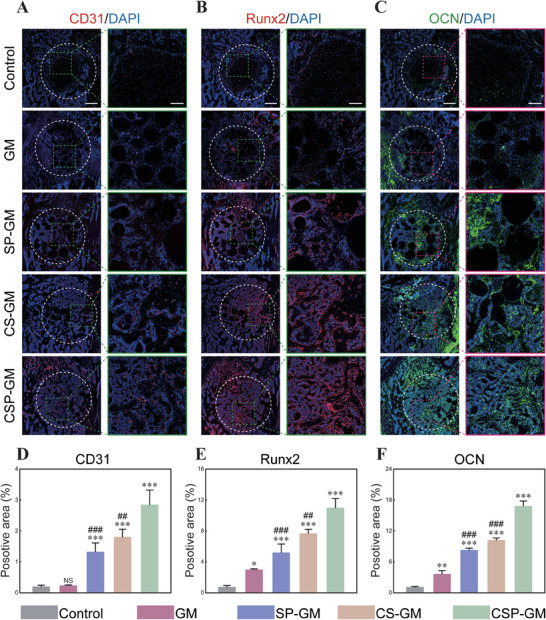
Immunofluorescent staining of femoral condyle in rats for angiogenesis and osteogenesis. Immunofluorescence staining of A) CD31, B) Runx2, and C) OCN expression in defect areas at 4 weeks postoperative (Scale bar, 500 µm and 200 µm for left and right magnified images, respectively). Quantitative analysis of positive areas of D) CD31, E) Runx2, and F) OCN (n = 3). NS: no significance, ^*^ *p* < 0.05, ^**^ *p* < 0.01, and ^***^ *p* < 0.001 compared with the Control group; ^#^ *p* < 0.05, ^##^ *p* < 0.01, and ^###^ *p* < 0.001 compared with the CSP‐GM group.

Early angiogenesis is an important driver for bone repair. Immunofluorescence staining for platelet‐endothelial cell adhesion molecule‐1 (PECAM‐1/CD31) revealed limited blood vessel invasions in control and GM groups, while a significant increase in positive CD31 expression was observed in both peripheral and internal regions of newly formed bone in the CSP‐GM group, surpassing the levels in the other groups (Figure 10A,D). Furthermore, immunofluorescence staining of Runx2 and OCN was employed to assess the composite oxygen‐generating system's capacity to promote osteogenesis during the bone repair progress. As showed in Figure 10B,C, the expression levels of osteogenic markers in groups treated with composite microspheres were considerably higher than those in the control and GA groups. Quantitative analysis revealed the strongest immunofluorescence signal for both Runx2 and OCN expression in the CSP‐GM group, followed by CS‐GM and SP‐GM groups (Figure 10E,F). These results strongly suggested that the oxygen generating system, integrated with ROS‐scavenging properties, could modulate microenvironment and effectively promoted neovascularization and osteogenesis, thereby accelerating bone tissue repair. Moreover, H&E staining analysis revealed no obvious damage to typical organs post‐implantation with different materials, confirming the excellent biocompatibility and biosafety that the hydrogel microspheres irrespective of nanoparticle addition (Figure S19, Supporting Information).

## 3 Conclusion

In summary, this implantable multifunctional micro‐oxygen reservoir was crafted by integrating hydrogel microspheres and composite oxygenating nanoparticles with broad‐spectrum ROS‐scavenging activity. It exerted multi‐hierarchy antioxidant activity and prolonged oxygen release behavior, effectively modulating the adverse hypoxia/oxidative stress microenvironment and preserving the regenerative activities of reparative cells. Furthermore, the ECM‐mimetic micro/nano‐structure of hydrogel microspheres provided structural support for cell growth and adhesion, promoting neovascularization and osseointegration, thereby resulting in enhanced tissue repair effects. In vivo results further confirmed the efficacy of composite microspheres in alleviating hypoxia‐oxidative stress, enhancing antioxidant activity, and promoting multiple regenerative processes including vascularization and osteogenesis. Together, the organic integration of optimized functional nanoparticles and micro/nano‐structured porous microspheres demonstrated a synergistic role in regulating osteogenic microenvironment and promoting vascularized bone regeneration. Our pro‐regeneration strategy provided valuable insights into the design and development of biomaterials for the treatment of complex bone defects.

## Conflict of Interest

The authors declare no conflict of interest.

## Author Contributions

M.R., J.M., H.W., and Y.H. contributed equally to this work. M.R., J.M., and H.H. synthesized the materials and performed materials characterization, performed in vitro and in vivo experiments. H.S. and Y.Y. assisted in in vivo experiments. T.M. and K.R. provided assistant in histology analysis. M.R., J.M., and H.W. analyzed the data and wrote the manuscript with help from all authors. W.C., Q.S., and H.Y. directed and supervised the study. All authors discussed the results and commented on the manuscript.

## Supporting information



Supporting Information

## Data Availability

The data that support the findings of this study are available from the corresponding author upon reasonable request.
