# Supplementary material for: Implantable Multifunctional Micro‐Oxygen Reservoir System for Promoting Vascular‐Osteogenesis via Remodeling Regenerative Microenvironment
Source: Adv Sci (Weinh). 2024 Nov 26;12(3):2409636. doi: 10.1002/advs.202409636 (PMC11744641; doi:10.1002/advs.202409636)
Supplement: Supplementary file 1 — Supporting Information [file ADVS-12-2409636-s001.docx]

**Supplementary Materials**

**Implantable Multifunctional Micro-Oxygen Reservoir System for Promoting Vascular-osteogenesis *via* Remodeling Regenerative Microenvironment**

***Min Rui^†^, Jiannan Mao^†^, Hongshuai Wu^†^, Yujian Hui^†^, Hao Shen, Yilin Yang, Tao Ma, Kewei Ren, Juan Wang, Wenguo Cui*, Qin Shi^*^, Huilin Yang^*^***

**M. Rui, J. Mao, Y. Hui, H. Shen, Y. Yang, Q. Shi, H. Yang**

Department of Orthopaedics, The First Affiliated Hospital of Soochow University, Orthopaedic Institute of Soochow University, 899 Pinghai Road, Suzhou, Jiangsu 215031, P. R. China

E-mail: [shiqin@suda.edu.cn](mailto:shiqin@suda.edu.cn) (Q. Shi), [suzhouspine@163.com](mailto:suzhouspine@163.com) (H. Yang)

**M. Rui, J. Mao, H. Wu, Y. Hui, K. Ren**

Department of Orthopaedics; Wuxi Key Laboratory of Biomaterials for Clinical Application, Department of Central Laboratory, Jiangyin Clinical College of Xuzhou Medical University, No.163 Shoushan Road, Jiangyin, Jiangsu 214400, P. R. China

**T. Ma**

Department of Orthopedics, The First Affiliated Hospital of Wannan Medical College, Yijishan Hospital, No. 2, Zhe Shan Xi Road, Wuhu, Anhui 241001, P. R. China

1. Wang, W. Cui

Department of Orthopaedics, Shanghai Key Laboratory for Prevention and Treatment of Bone and Joint Diseases, Shanghai Institute of Traumatology and Orthopaedics, Ruijin Hospital, Shanghai Jiao Tong University School of Medicine, 197 Ruijin 2nd Road, Shanghai 200025, P. R. China

mail: [wgcui80@hotmail.com](mailto:wgcui80@hotmail.com) (W.Cui)

**Supplementary experimental section**

**Synthesis of Calcium Peroxide Nanoparticles:** Calcium peroxide nanoparticles (CaO_2_ NPs) were synthesized via a hydrolysis-precipitation procedure.^[1]^ Briefly, calcium chloride dihydrate (CaCl_2_·2H_2_O, 300 mg) was initially dissolved in 3 mL of double distilled water. Afterwards, 2 mL of ammonium hydroxide (NH_3_.H_2_O, 1 M) and 12 mL of Polyethylene glycol (PEG-200) were added into the solution under magnetic stirring to facilitate the reaction. Thereafter, 1.5 mL of hydrogen peroxide (H_2_O_2_, 30% w/w) solution was incrementally added to the stirring mixture at a controlled rate of 0.04 mL min ^-1^ via a programmable infusion pump. The mixed was continuously stirred for 2 hours to obtain a yellowish solution. Subsequently, a sodium hydroxide solution (NaOH, 0.1 M) was gradually introduced under continuous stirring to adjust the pH to 11.5, precipitating a white suspension. The suspension was then centrifuged at 12,000 revolutions per minute (rpm) for 15 min to isolate the CaO_2_ nanoparticles. The obtained particles were sequentially purified by sequential washing with NaOH solution and absolute ethyl alcohol, concluding with vacuum drying.

**SiO_2_ Coating:** 2 mg of CaO_2_ was initially dissolved into 25 mL of absolute ethyl alcohol solution, followed by sonication for 10 min to ensure a homogeneous suspension. Subsequently, 1 mL of NH_3_.H_2_O (28 wt.%) was introduced, and the mixture was stirred at ambient temperature for 1 hour. Thereafter, a mixture of 9.8 mL of ethanol and 0.2 mL of ethyl silicate (TEOS) was incrementally added dropwise over a period of 3 hours using an infusion pump, while maintaining continuous stirring for a duration of 12 hours to promote even silica layer formation. The resulting products were collected via centrifugation at 12,000 rpm for 10 min, followed by ethanol wash for 3 times to remove any unreacted precursors. The resulting products were stored after drying.

**PDA Coating:** 50mg of CaO_2_@SiO_2_ nanoparticles were added into 25 mL of Tris-HCl buffer solution (10 mM, pH 8.5), followed by ultrasonic dispersion for 5 min. Subsequently, 25 mg of dopamine hydrochloride was added and the mixture was continuously stirred for 6 h to facilitate. Afterward, the PDA-coated particles were collected by centrifugation and washed with deionized water three times to remove any unreacted material. Finally, the CaO_2_@SiO_2_@PDA (CSP) nanoparticles were stored at 4°C for future use.

**Amino-modification of CaO_2_@SiO_2_ Nanoparticles:** 50 mg of CaO_2_@SiO_2_ nanoparticles were dispersed in 20 mL of ethanol, followed by the addition of 0.5 mL of (3-aminopropyl) trimethoxy silane (APTES). The resulting solution was then refluxed at 80 ℃ for 12 hours, yielding amino-modification of the CaO_2_@SiO_2_ particles. The obtained nanoparticles were centrifuged and thoroughly washed with ethanol three times. Finally, the products were stored after being dried in a vacuum chamber.

**Preparation of GelMA:** Gelatin methacrylate anhydride (GelMA) was synthesized following the methods previously described. Initially, gelatin (20 g) was dissolved in 200 mL of Phosphate-Buffered Saline (PBS) and stirred at 60 ℃ for 1 hour, ensuring the complete dissolution to obtain a 10% (w/v) homogeneous gelatin solution. Then, under light-proof conditions, 16 mL of methacrylic anhydride (MA) was introduced dropwise at a rate of 0.25 mL min ^-1^ using an infusion pump. The mixture was continuously stirred at 60 ℃ for 2 hours. After that, 800 mL of preheated PBS was added to terminate the reaction, and stirring was continued for a further 15 min. The solution then underwent dialysis using dialysis bags (12-14 kDa, MWCO) for a period of 2 weeks. Finally, the purified GelMA solution was lyophilized and stored at -20 ℃ for future utilization.

**GelMA Hydrogel Microspheres Fabrication:** The GelMA hydrogel microspheres were fabricated using a water-in-oil microfluidic technique. The programmable microfluidic device comprises an inner dispersed phase and an outer continuous phase. Specifically, the inner dispersed phase was composed of 7% (w/v) GelMA solution containing 0.5% (w/v) photoinitiator, while the outer phase contained mineral oil with 10% (w/w) Span 80. The GelMA droplets within the internal aqueous phase were generated through the coaxial shearing of continuous oil phase. By precisely adjusting flow rate parameters for both phases, a continuous stream of uniform, monodisperse spherical microdroplets was successfully generated. The microspheres were then synchronously polymerized under ultraviolet (UV) irradiation. Finally, the uniform GelMA microspheres were collected and purified using 75% ethanol and PBS, followed by freeze-drying to yield porous hydrogel microspheres.

**Grafting of Nanoparticles:** The lyophilized microspheres, along with 16 mg of EDC and 24 mg of NHS, were sequentially added to 5 mL of MES buffer (pH 6.0). After incubation at 37 °C for 15 min, the activated microspheres were collected via centrifugation. Thereafter, the prepared nanoparticles were evenly dispersed in 10 mL of deionized water, followed by the addition of activated microspheres. After stirring the mixture overnight at 37°C, and the modified microspheres obtained were washed and subjected to freeze-drying for further utilization.

**Material Characterization:** The particle sizes and zeta potential were assessed using dynamic light scattering (DLS, Malvern Zetasizer Nano ZS-90, UK). And the morphology of nanoparticles was observed by transmission electron microscopy (TEM, Hitachi HT7700, Japan). The elemental composition was analyzed by X-ray photoelectron spectroscopy (XPS, Thermo Fisher Scientific, US), while the phase composition was determined via X-ray diffraction (XRD, Bruker D8, German). Fourier transform infrared (FTIR) spectra were recorded using FTIR spectrometer (Thermo Fisher Nicolet 6700, US). The morphology and surface conditions of different microspheres were scrutinized through scanning electron microscopy (SEM, Hitachi S 4800, Japan), and the element distribution was simultaneously tested with X-ray spectroscopy (EDS) mapping to confirm the successful binding of microspheres and nanoparticles. Moreover, the surface roughness was examined by atomic force microscopy (AFM, Bruker Dimension FastScan, Germany).

**ABTS Radical Scavenging Assay:** The overall antioxidant capacity of CaO_2_@SiO_2_@PDA NPs was initially investigated using the 2,2’-azino-bis-3-ethylbenzothiazoline-6-sulfonic acid (ABTS) radical scavenging assay. The ABTS was converted to its radical form ABTS+•, by reacting with potassium persulfate solution. Subsequently, different concentrations of NPs (20, 40, 60, 80 and 100 μg mL^-1^) were introduced to the ABTS+• solution and allowed to incubate for 10 min. Finally, the absorbance at 734 nm of the resultant solutions was measured.

**O_2_^•-^ Radical Scavenging Assay:** The superoxide anion (O_2_^•-^) scavenging effects of the CSP NPs were subsequently evaluated using the pyrogallol autoxidation method. In this process, pyrogallol was reacted with NPs at concentrations ranging from 0 to 80 μg mL^−1^ for a duration of 30 min in darkness. Post the reaction period, the mixture was subjected to centrifugation, and the absorbance of the supernatant at 320 nm was measured.

**In vitro degradation of microspheres:** In the in vitro degradation assay, the freeze-dried microsphere samples were weighted and subsequently transferred into microcentrifuge tubes. Then, 1 mL of PBS solution containing 2 U/mL of collagenases was added to each tube. The tubes were placed in a shake at 37 °C, and the incubation medium was refreshed daily. At pre-defined intervals, the microspheres were collected, washed with sterile deionized water and reweighed after drying. The residual weight (%) was calculated with the following formula (**Equation 1**) based on the residual dry weight (W_1_) and the initial dry weight (W_0_):

Residual weight = $\frac{W1}{W0}$ $\times$100% (1)

**H_2_O_2_ Scavenging Assay:** The hydrogen peroxide (H_2_O_2_) scavenging efficiency of CSP NPs was evaluated by measuring the inhibition of indigo carmine oxidation.^[2]^ A solution containing H_2_O_2_ (5 mM) and indigo carmine (75 μM) was prepared and incubated with varying concentrations of NPs in darkness for 24 hours. Post incubation, the mixture was centrifuged, and the absorbance of the supernatant at 610 nm was measured.

**Measurement of Oxygen Release:** To evaluate the oxygen generation capacity of the biomaterials, the prepared nanoparticles and microspheres were immersed in phosphate-buffered saline (PBS, pH 7.4) and incubated at 37 ◦C. A control group was established using PBS devoid of any test materials. At different predefined time-points, the dissolved oxygen concentrations were measured using a dissolved oxygen probe (Smart Sensor, China).

**RNA Sequencing and Analysis:** The gene expression profiles of three groups were investigated by RNA sequencing: BMSCs cultured under normoxic conditions, those cultured in a hypoxic environment, and those cultured in hypoxia with the addition of CSP- GelMA. After culturing for 24 hours, total RNA was extracted using the TRIzol reagent, and RNA sequencing was performed by OE Biotech Co. Differentially expressed genes (DEGs) were identified with a statistical significance threshold of p-value ＜0.05 and a fold change ＞2.0. Then the DEGs were subjected to Gene Ontology (GO) and Kyoto Encyclopedia of Genes and Genomes (KEGG) enrichment analysis.

**Cell Viability and Proliferation Assay:** After co-incubation of BMSCs with the microspheres under hypoxic conditions using the Anaeropack anaerobic system for the specified duration, cell proliferation was assessed by live/dead staining and Cell Counting Kit-8 (CCK-8) assay. The viability of BMSCs on the microspheres was evaluated at 3 and 5 days of co-culturing using live/dead cell staining following the manufacturer’s protocols. Afterwards, the morphology and live/dead cells distribution on the microspheres were observed using a fluorescence microscope (Carl Zeiss, Germany). Additionally, the CCK-8 assay was utilized to evaluate the proliferation of cells on microspheres. Moreover, BMSCs (2×10^4^/well) and microspheres were respectively seeded in the lower and upper chambers of a Transwell insert and co-cultured in the Anaeropack anaerobic system. At intervals of 1, 3 and 5 days, the culture medium containing 10% of CCK-8 reagent (Dojindo, Japan) were added to the cell-microspheres co-culture system. After incubation for 4 h, 100 μL of the supernatant was transferred into a 96-well plate and the optical density (OD) value at 450 nm was measured using a microplate reader (BioTek, USA).

**Cytoskeleton Staining:** The morphology and adhesion of BMSCs on microspheres were assessed by Phalloidin/DAPI staining. After incubation for 3 and 7 days under hypoxia conditions, the cell-microspheres constructs were washed with PBS and fixed with 4% paraformaldehyde for 30 min. The samples were then perforated with 0.3% Triton X-100 for 30 min and subsequently blocked in a 5% bovine serum albumin (BSA) solution at 4 °C overnight. Subsequently, the samples were incubated in a phalloidin solution and further stained with DAPI. Finally, the stained cell-microspheres were observed using a fluorescence microscope, with F-actin filaments and nuclei being labeled in red and blue, respectively.

**Determination of Intracellular ROS:** To evaluate the intracellular reactive oxygen species (ROS) levels, BMSCs and microspheres were respectively seeded in the lower and upper chambers of a Transwell insert for 24 h within an Anaeropack anaerobic system. A control group, devoid of microspheres, was also established for comparison. Subsequently, BMSCs were incubated with the fluorescent probe DCFH-DA in a cell incubator at 37 ℃ for 0.5 h and then washed three times with PBS. Finally, they were observed under a fluorescence microscope (Carl Zeiss, Gremany) and analyzed using flow cytometry.

**Determination of Mitochondrial ROS:** Mitochondrial ROS was assessed using MitoSox probes (MedChemExpress, USA). BMSCs and microspheres were respectively seeded in the bottom and upper chamber of a Transwell insert for 24 h in the Anaeropack anaerobic system. To specifically target the mitochondria in BMSCs, the cells were initially incubated in a pre-warmed 37 ◦C working staining solution of MitoTracker Green (100 nM) for 30 min. After incubation, the cells were washed with PBS and then further incubated with a MitoSOX Red (5 μM) working solution for 20 min before being observed under a confocal microscope (Carl Zeiss, Gremany).

**Mitochondrial Membrane Potential Assay:** The mitochondrial membrane potential (MMP) of BMSCs was evaluated using the JC-1 MMP assay kit (MedChemExpress, USA). BMSCs and microspheres were respectively seeded in the lower and upper chambers of a Transwell insert in the Anaeropack anaerobic system. After a 24-hour co-incubation, the cells were incubated with the JC-1 working solution for 20 min at 37 °C, then washed with PBS and observed under a fluorescence microscope (Carl Zeiss, Gremany). The MMP, indicated by the ratio of red to green JC-1 fluorescence intensity, was quantified using ImageJ software.

**Wound Healing Assay:** Human umbilical vein endothelial cells (HUVECs, 2×10^5^ cells/well) were seeded in the lower chamber of 12-well plates with Transwell insert. After reaching approximately 90% confluence, a linear scratch was created on the monolayer cells using a sterile 100 μL pipette tip. The well was then gently washed with PBS to remove floating cells. Control (HUVECs only) and cells with GelMA or composite microspheres placed into the upper chamber were cultured in fresh serum-free medium. After co-culturing in an Anaeropack anaerobic system for 24 h, the wound healing status was captured using microscopy and quantified using Image J software.

**Tube Formation Assay:** The endothelial tube formation assay was performed to assess the angiogenesis of HUVECs from each group. Following the manufacturer’s protocol, HUVECs (3×10^4^ cells/well) were seeded onto the Matrigel-precoated 24-well plates with Transwell insert, and microspheres were placed in the upper chamber, followed by co-incubation in an anaerobic system to form a vascular-like network structure. After 12 and 24 h of incubation, tube formation on the Matrigel surface was observed using an optical microscope respectively, and the results at the 24-hour time point were subsequently analyzed.

**ALP Activity Assay:** The alkaline phosphatase (ALP) activity of BMSCs was assessed through ALP staining and an ALP activity quantification kit. After 7 days of co-culture and osteogenic induction in an Anaeropack anaerobic system, the cell-microspheres were fixed by 4% paraformaldehyde solution, washed with PBS, and then incubated with the ALP staining solution. Following the same preparatory treatment, BMSCs and different microspheres were further seeded on the lower and upper layers of Transwell insert respectively. After 7 days, the cells underwent ALP staining and observed under an optical microscope. Moreover, the ALP activity was quantified with an ALP Assay Kit (Beyotime Biotech, China), following the manufacturer’s protocol.

**Alizarin Red Staining:** The calcium deposition effect in different groups was evaluated by Alizarin Red S (ARS) staining. BMSCs were either directly seeded onto microspheres or cultured in separate compartments of Transwell insert, with BMSCs seeded in the lower layer and microspheres in the upper layer. After co-cultured in osteogenic medium for 14 days, the cell-microspheres and the individual cells were washed with PBS and fixed by 4% paraformaldehyde solution for 30 min, followed by staining with the ARS staining kit (Cyagen, China). The calcium nodules were observed by microscope and subsequently dissolved in perchloric acid, and the optical density (OD) values at 420 nm were measured.

**Immunofluorescence Staining:** The expression of osteogenic markers in cells cultured on microspheres was determined using immunofluorescence staining analysis. The microspheres and cells were co-cultured under hypoxic conditions as described above. Following osteo-inductive co-culture for 7 and 14 days, the cell-microspheres were fixed with 4% paraformaldehyde, permeabilized with 0.1% Triton X-100, blocked with 5% BSA, and then incubated with primary antibodies specific for Runx2 (7 days) and OCN (14 days) at 4 ℃ overnight, followed by further treatment with appropriate secondary antibodies. After washing with PBS, the samples were counterstained with phalloidin and DAPI. Finally, the stained samples were observed and analyzed using confocal microscopy (Carl Zeiss, Germany) and ImageJ software.

**Quantitative real-time polymerase chain reaction PCR (qRT-PCR):** After co-culturing with microspheres in a Transwell insert within the Anaeropack anaerobic system, total RNA was extracted from BMSCs using the Trizol reagent at predetermined intervals. The expression of relevant genes was then evaluated using a quantitative Realreal-time polymerase chain reaction PCR (qRT-PCR) assay, with Glyceraldehyde-3-phosphate dehydrogenase (*GAPDH*) used as the internal reference gene. And the relative expression levels were calculated using the 2^-ΔΔCt^ method. The primer sequences utilized were summarized in Table S1(Supporting Information).

**Western blot assays:** Total proteins were extracted using radioimmunoprecipitation assay (RIPA) lysis buffer containing protease inhibitor and phosphatase inhibitor (Beyotime, China). Protein concentration was quantified using a bicinchoninic acid assay (BCA) protein quantification kit (Solarbio, China). Equal amounts of protein samples were separated with sodium dodecyl sulfate-polyacrylamide gel electrophoresis (SDS-PAGE) and transferred to polyvinylidene fluoride (PVDF) membranes. After blocking, the membranes were incubated with the primary antibody including anti-Nrf2 antibody (SAB, USA), anti-HO-1 antibody (SAB, USA), anti-SOD2 antibody (SAB, USA), anti-GAPDH antibody (SAB, USA) at 4 °C overnight and further incubated with the corresponding secondary antibody at room temperature for 1 h. The protein bands were visualized using an enhanced chemiluminescence kit (Beyotime, China) on a chemiluminescent imaging system (Monad, China). Finally, the relative density of the protein bands was analyzed using ImageJ software.

**Rat Femoral Condylar Defect Model Establishment:** Male Sprague-Dawley (SD) rats, weighing 300-350 g, were utilized for the experimental procedures. All protocols involving animals were reviewed and approved by the Animal Ethics Committee of Soochow University (Approval code: SUDA20230911A04). SD rats were randomly assigned to five distinct experimental groups: Control group (simple surgery), GM group (surgery + GM treatment), SP-GM group (surgery + SP-GM treatment), CS-GM group (surgery + SP-GM treatment), and CSP-GM group (surgery + CSP-GM treatment). A femoral condylar defect model was surgically created in the rats. Before operation, the rats were anesthetized using an intraperitoneal injection of 2% sodium pentobarbital (2.5 mL/kg). The lateral femoral condyle was exposed via blunt separation of intermuscular space, with protection of the adjacent blood vessels and soft tissues. The femoral condylar double-cortex was then drilled to create a defect with a 3-mm diameter bit for penetrating both cortices. After being cleaned with aseptic saline solution, the defects were filled with the respective sterilized microspheres, and the surgical incision was meticulously closed in layers. A control group was established with no implanted materials. After surgery, all rats were administered daily intramuscular injections of penicillin for 3 days to prevent infection.

**Detection of in vivo ROS Level:** The in vivo ROS levels in the bone defect area were detected one week post-surgery using ROS Brite 700 (AAT Bioquest, USA). The rats were anesthetized with isoflurane and then locally injected with in vivo imaging ROS Brite 700 probes (100 μM in Hanks with 20 mM Hepes buffer, HHBS) into the femoral condyle defect site. Finally, the rats were observed with an in vivo imaging system (PerkinElmer, Inc., USA) and bioluminescence images were captured.

**Radiological Assessment:** At 2 and 4 weeks post-surgery, femoral specimens of rats were collected, and subsequently fixed in a 10% formalin solution after euthanasia. These specimens were then subjected to micro-CT (SkyScan1176, Belgium) scanning with specific settings: 18 µm, 65 kV, 500 µA. Subsequently, three-dimensional (3D) reconstruction was conducted and bone morphometric parameters including bone mineral density (BMD), bone tissue volume/total tissue volume (BV/TV), trabecular thickness (Tb.Th) and trabecular separation (Tb.Sp) were measured and analyzed.

**Histological Analysis:** The femur specimens were fixed in a 4% paraformaldehyde solution for 48 hours, followed by decalcification using a 10% EDTA solution at room temperature for 30 days. Once decalcified, the femurs were embedded in paraffin and sectioned to a thickness of 5 μm along the sagittal axis. The obtained sections then subjected to hematoxylin-eosin (H&E) and Masson’s trichrome staining to analyze morphological differences in the defect area. Immunofluorescence staining for specific markers (Hif-1α, Nrf2, CD31, Runx2, and OCN) was performed to explore osteogenesis capacity, angiogenesis, and antioxidative effects in vivo. Additionally, to evaluate the biosafety of biomaterials in vivo, major viscera, including the heart, liver, spleen, lung, and kidney were collected and processed for H&E staining at 4 weeks post-implantation.

**Statistics Analysis:** All quantitative data (unless otherwise specified) were presented as the mean ± standard deviation (SD). The statistical analysis was performed using Origin 2018 software (Origin Lab Inc., USA) with One-way analysis of variance (ANOVA) and Tukey’s multiple comparison to identify the differences between groups. *P* < 0.05 (two-tailed) was considered to be statistically significant.

**Table S1.** Primers used in qRT-PCR

| Gene | Primer | Sequence |
| --- | --- | --- |
| *Nfe2l2* | Forward  Reverse | GCCTTCCTCTGCTGCCATTAGTC  TGCCTTCAGTGTGCTTCTGGTTG |
| *Hmox1* | Forward  Reverse | AGGAACACAAAGACCAGAG  CAGAGGTAGTATCTTGAACCAG |
| *Sod2* | Forward  Reverse | GAACCCAAAGGAGAGTTGC  CACAGCTGTCAGTTTCTCC |
| *Hif-1α* | Forward  Reverse | CCCTACTATGTCGCTTTCTTGG  GTTTCTGCTGCCTTGTATGGG |
| *Vegfa* | Forward  Reverse | CGGTGTGGTCTTTCGTCCTTCTTAG AGGGATGGGTTTGTCGTGTTTCTG |
| *Alp* | Forward  Reverse | CGTCTCCATGGTGGATTATGC  CCCAGGCACAGTGGTCAA |
| *Runx2* | Forward  Reverse | TCTTCCCAAAGCCAGAGC  TGCCATTCGAGGTGGTCG |
| *Col 1* | Forward  Reverse | CAGGCTGGTGTGATGGGATT  CCAAGGTCTCCAGGAACACC |
| *Ocn* | Forward  Reverse | AGGACCCTCTCTCTGCTCA  AACGGTGGTGCCATAGATGC |
| *GAPDH* | Forward  Reverse | AACTCCCATTCTTCCACC  TTGTCATACCAGGAAATGAG |

**Supplementary figures**

**
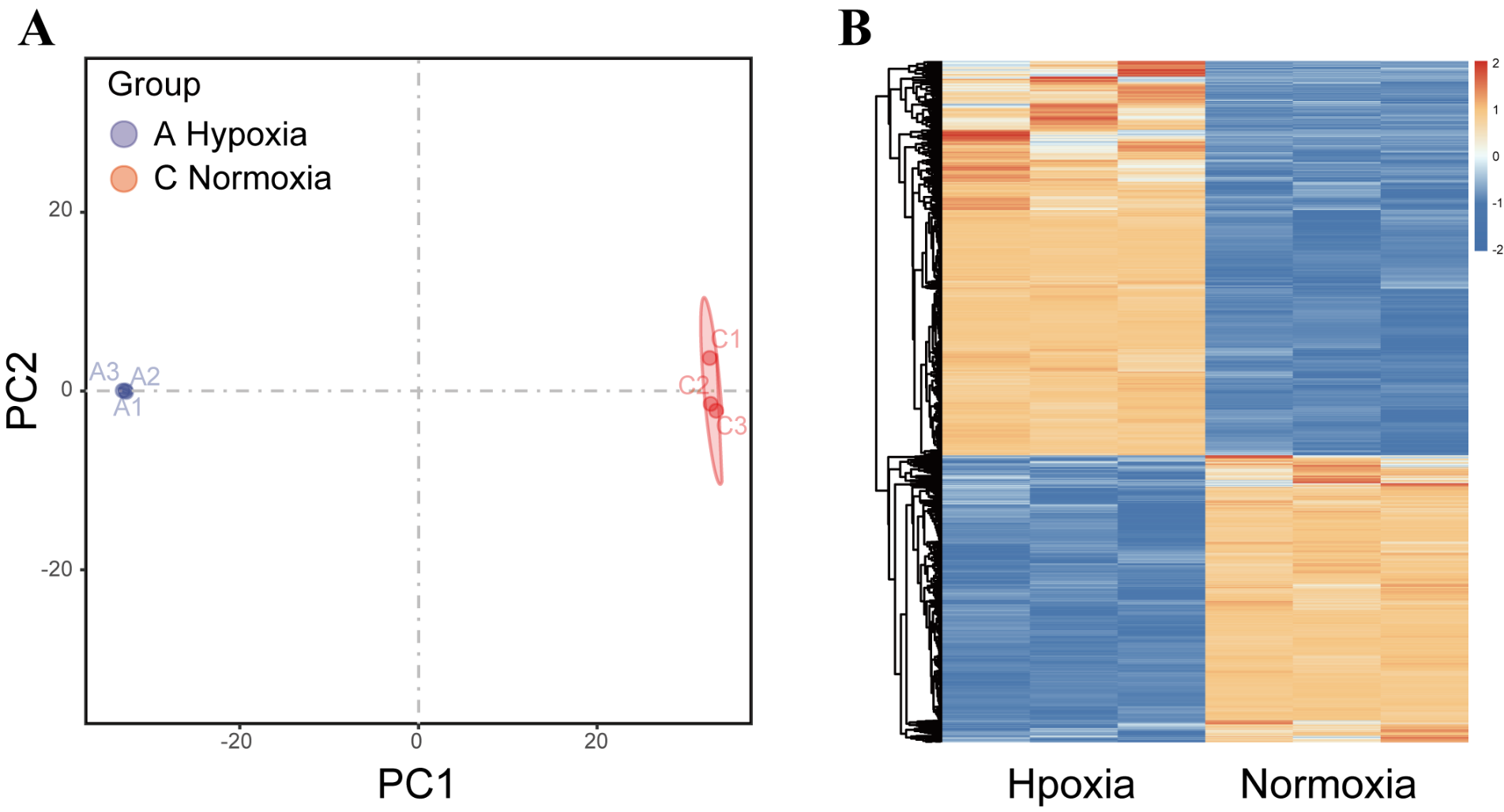
**

**Figure S1.** A) PCA of samples. B) Heatmap of DEGs.


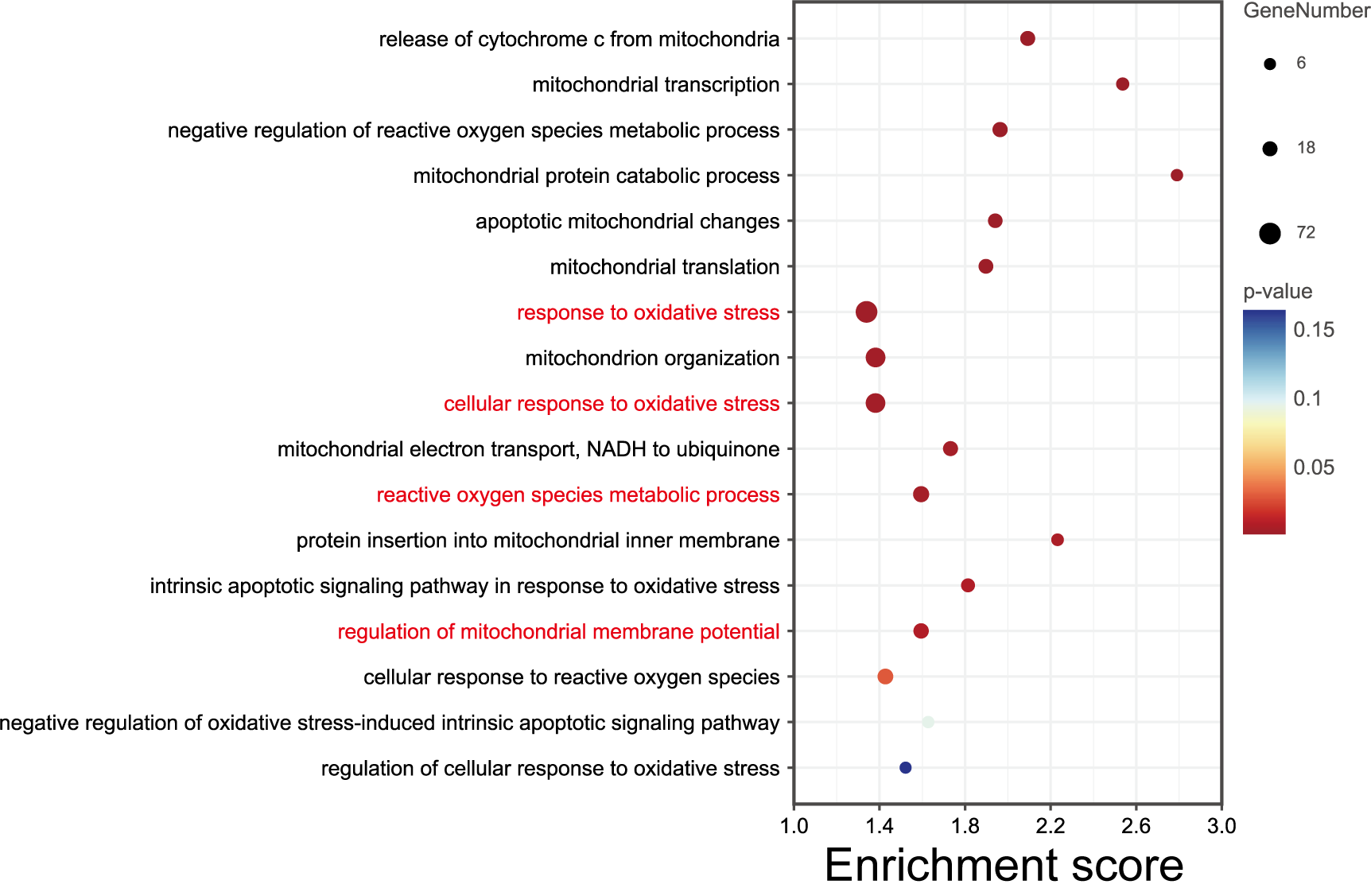


**Figure S2.** GO enrichment analysis of DEGs concerning mitochondria and oxidative stress.


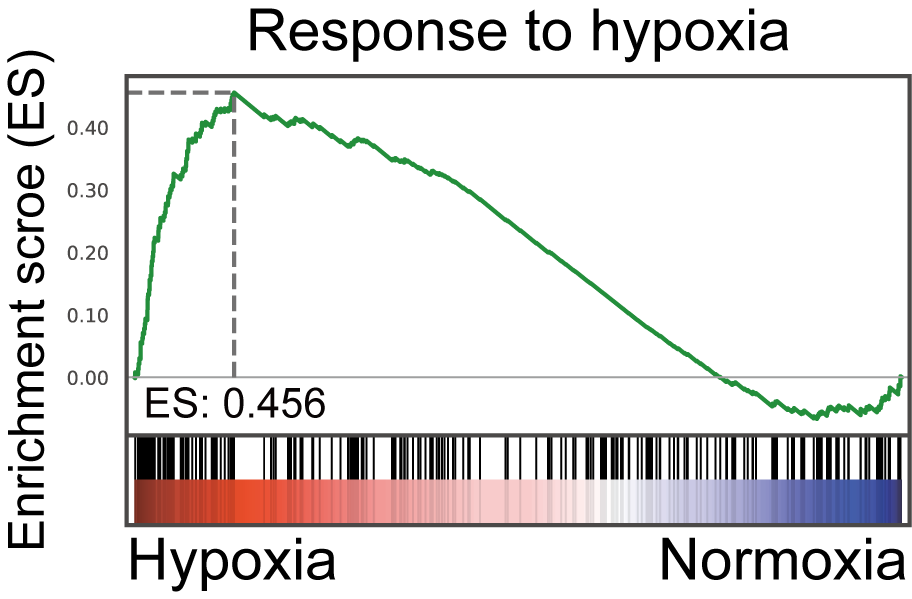


**Figure S3.** GSEA analysis of response to hypoxia.

**
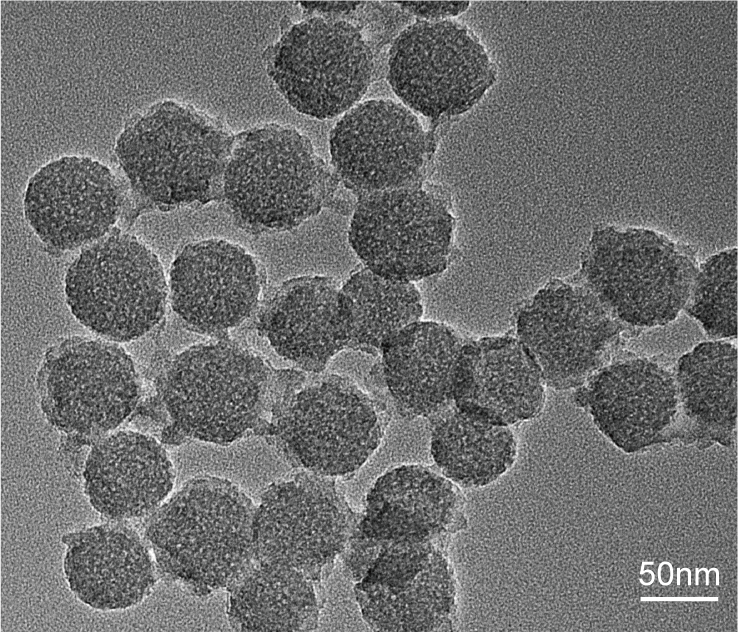
**

**Figure S4.** TEM images of SiO_2_@PDA (SP) nanoparticles (NPs).

**
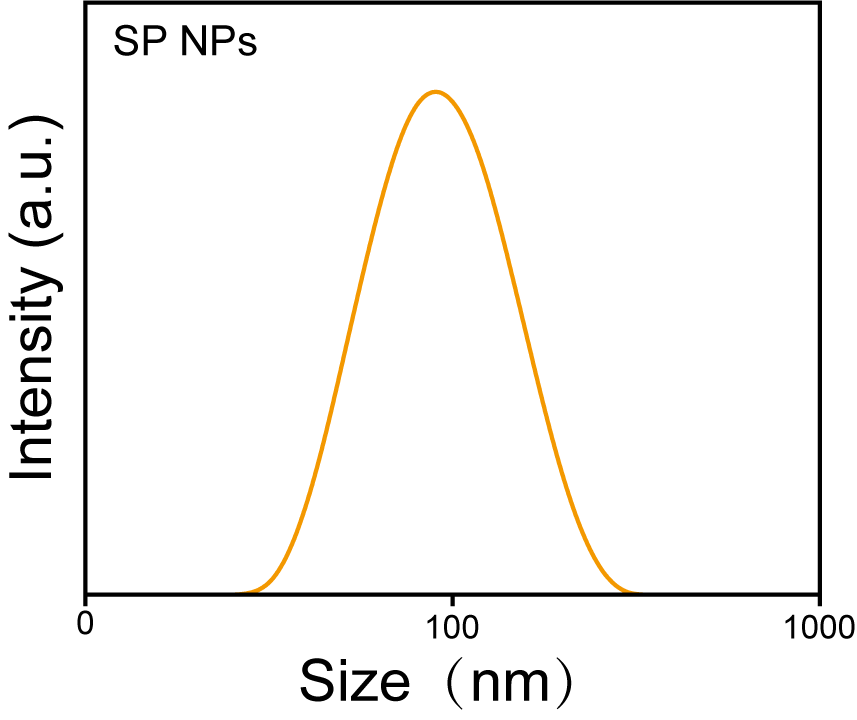
**

**Figure S5.** Particle size analysis of SP NPs.

**
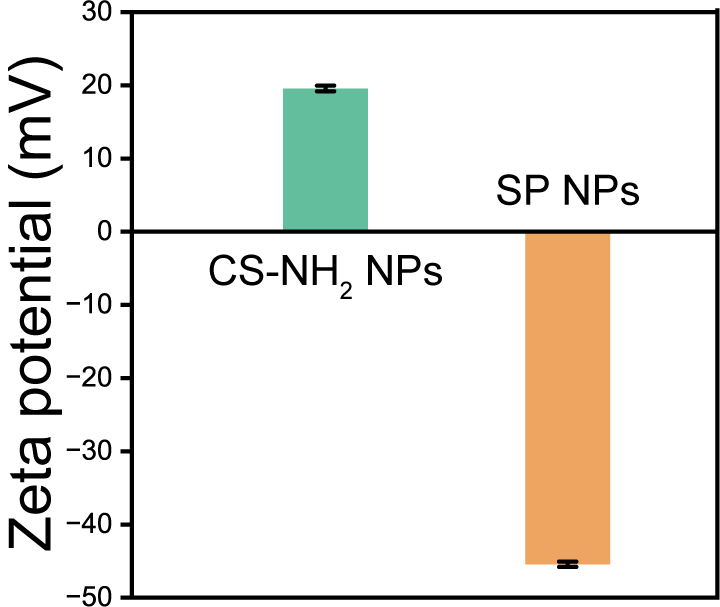
**

**Figure S6.** Zeta potential analysis of CaO_2_@SiO_2_-NH_2_ and SP NPs (n=3).

**
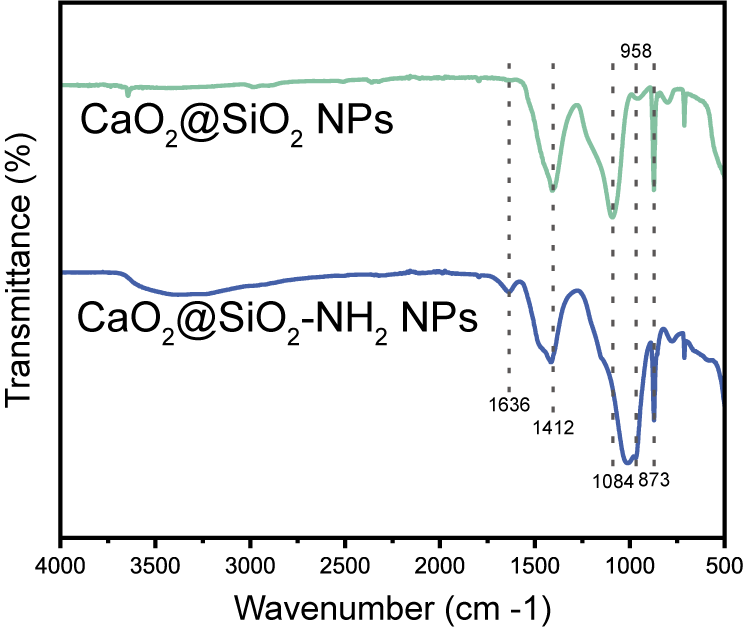
**

**Figure S7.** FTIR spectra of CaO_2_@SiO_2_ and CaO_2_@SiO_2_-NH_2_.

**
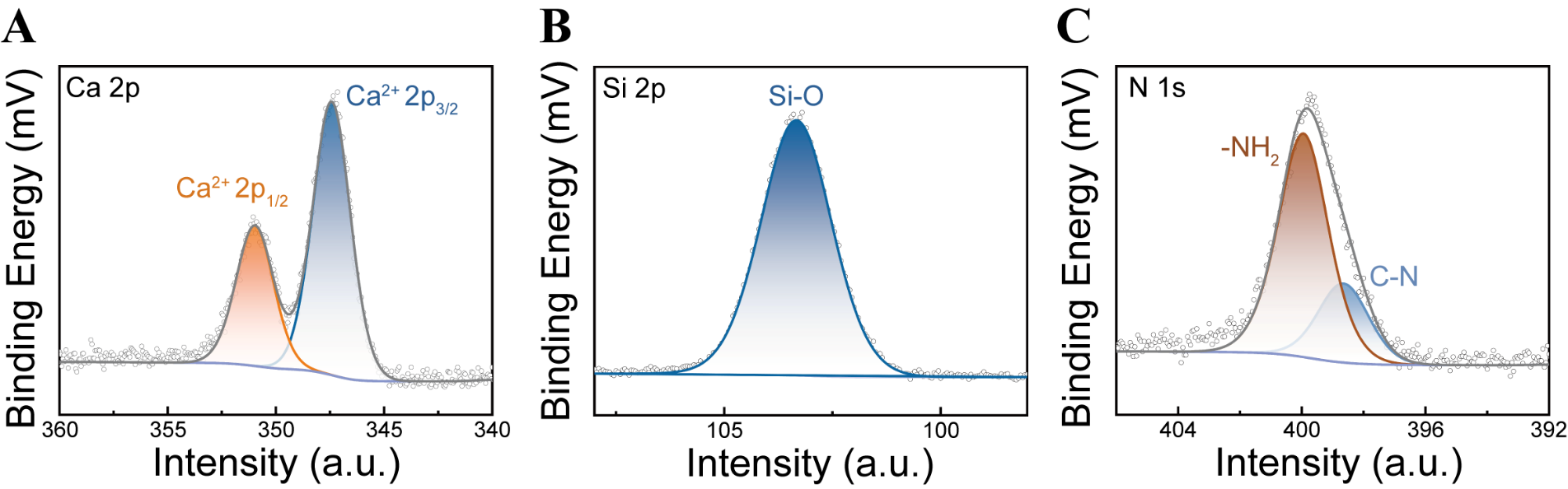
**

**Figure S8.** XPS high-resolution spectra of A) Ca 2p, B) Si 2p and C) N 1s signals for CaO_2_@SiO_2_@PDA.

**
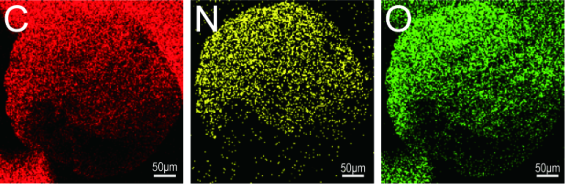
**

**Figure S9.** SEM mapping analysis of GelMA microsphere.


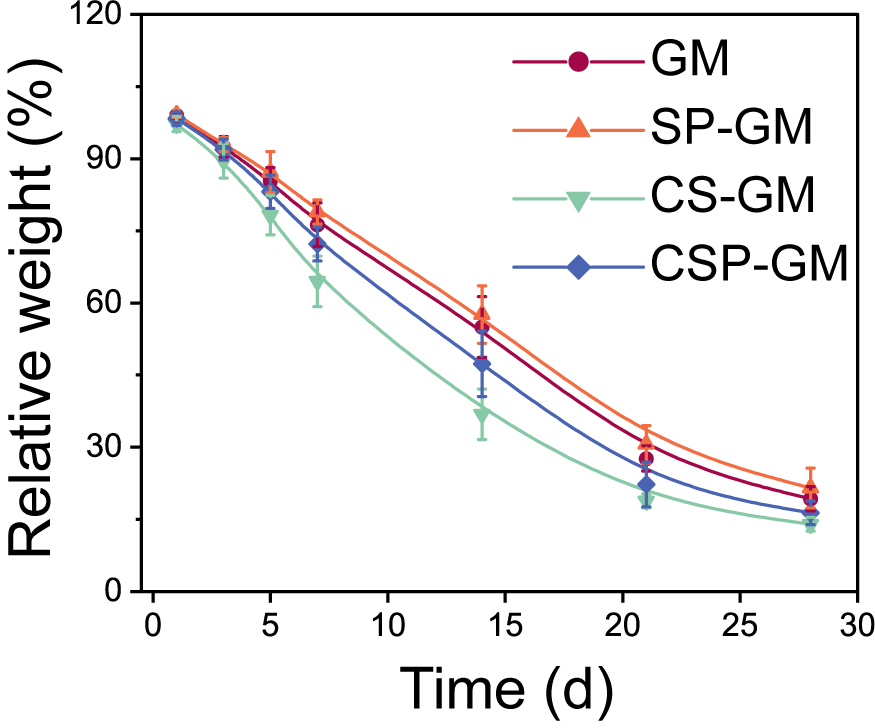


**Figure S10.** The degradation curve of microspheres in PBS solution containing 2 U/mL of collagenases (n=3).


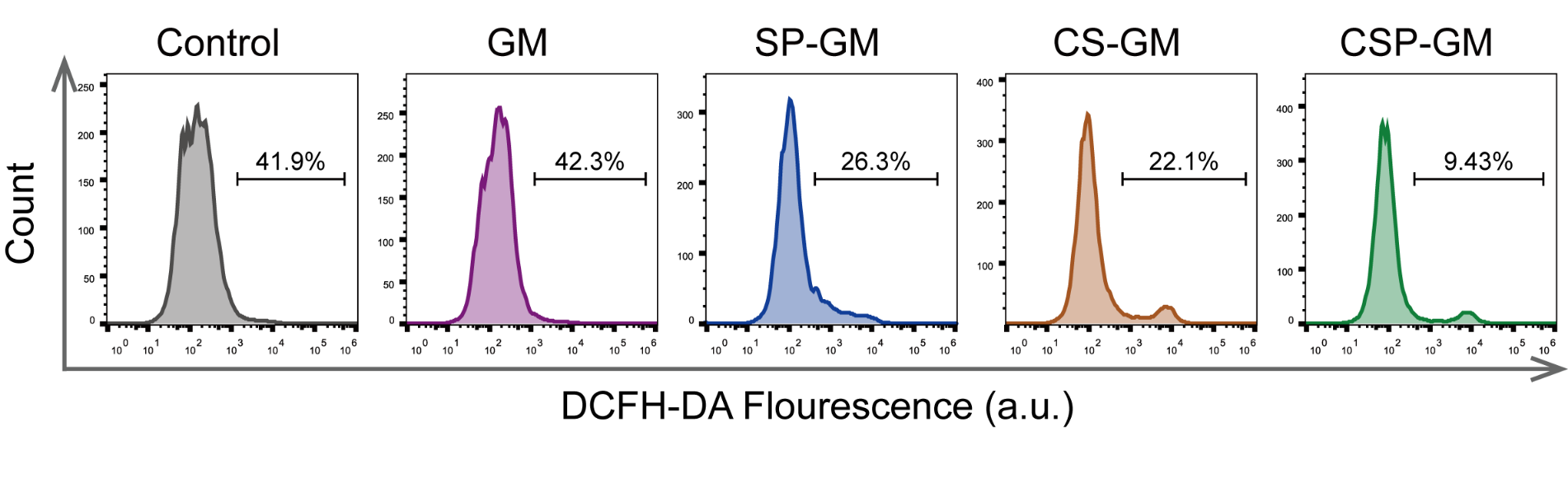
**Figure S11.** Flow cytometry analysis of ROS levels in BMSCs using DCFH-DA probe.

**
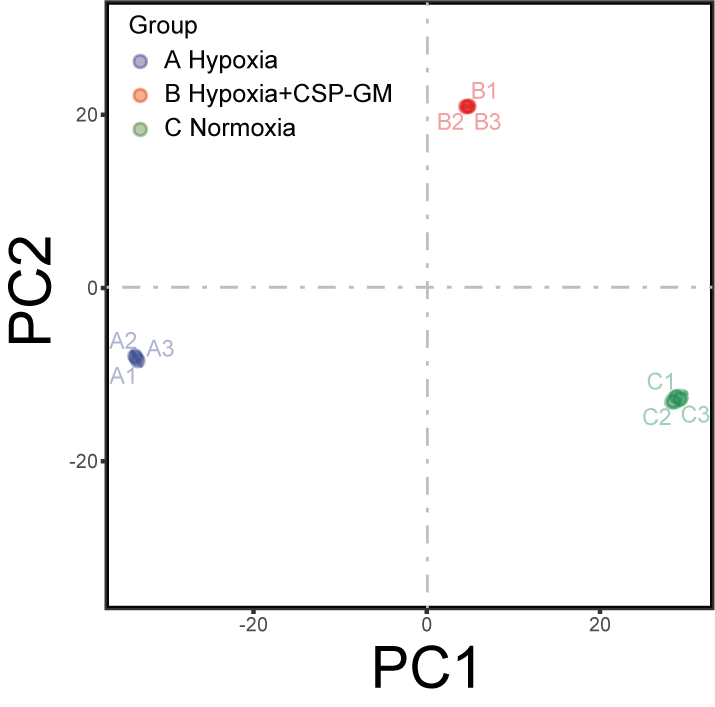
**

**Figure S12.** PCA of samples in hypoxia group, hypoxia+CSP-GM group, and normoxia group.

**
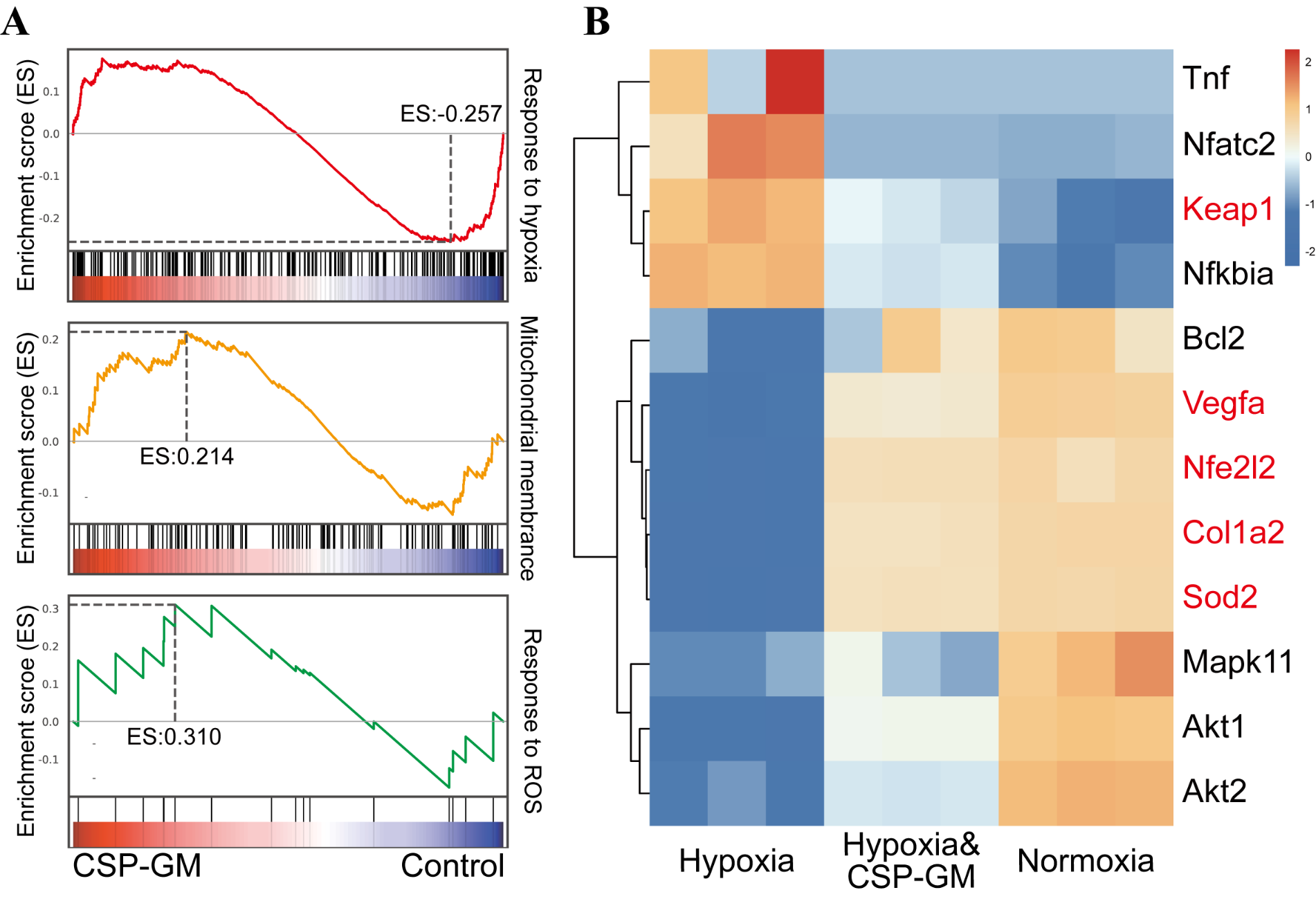
**

**Figure S13.** A) GSEA analysis of mitochondrial membrane and response to ROS in hypoxia conditions. B) Heat map of DEGs among three groups.


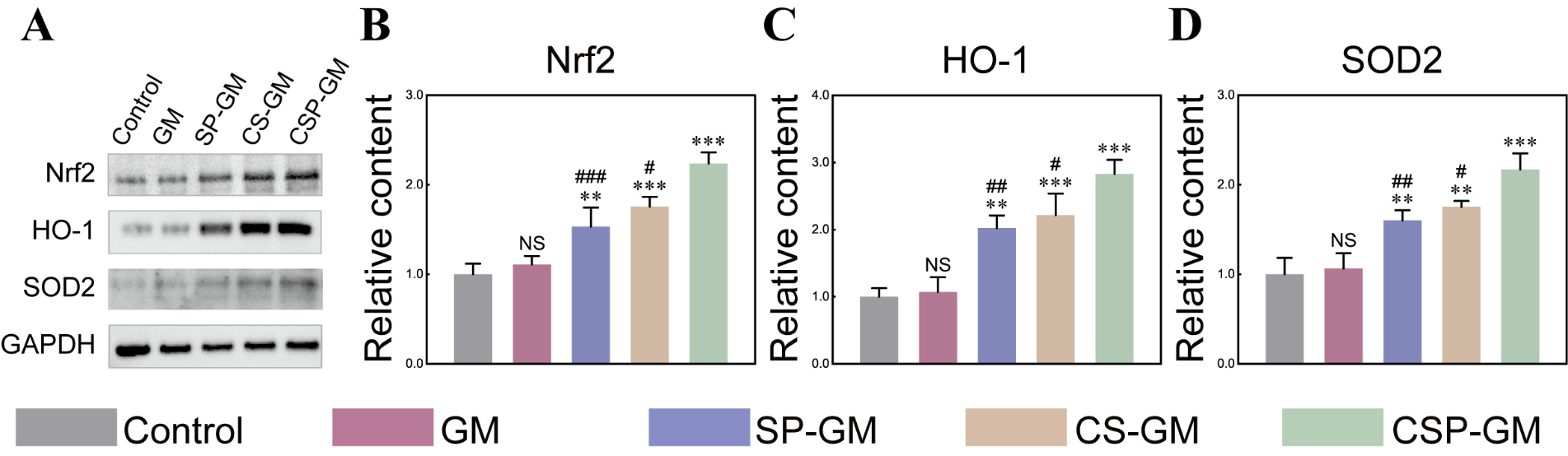


**Figure S14.** A) Western blot of Nrf2, HO-1 and SOD2 protein expression in BMSCs; B-D) Quantification analysis of Nrf2, HO-1 and SOD2 protein levels (n=3). NS: no significance, ^*^*P* < 0.05, ^**^*P* < 0.01, and ^***^*P* < 0.001 compared with the Control group; ^#^*P* < 0.05, ^##^*P* < 0.01, and ^###^*P* < 0.001 compared with the CSP-GM group.

**
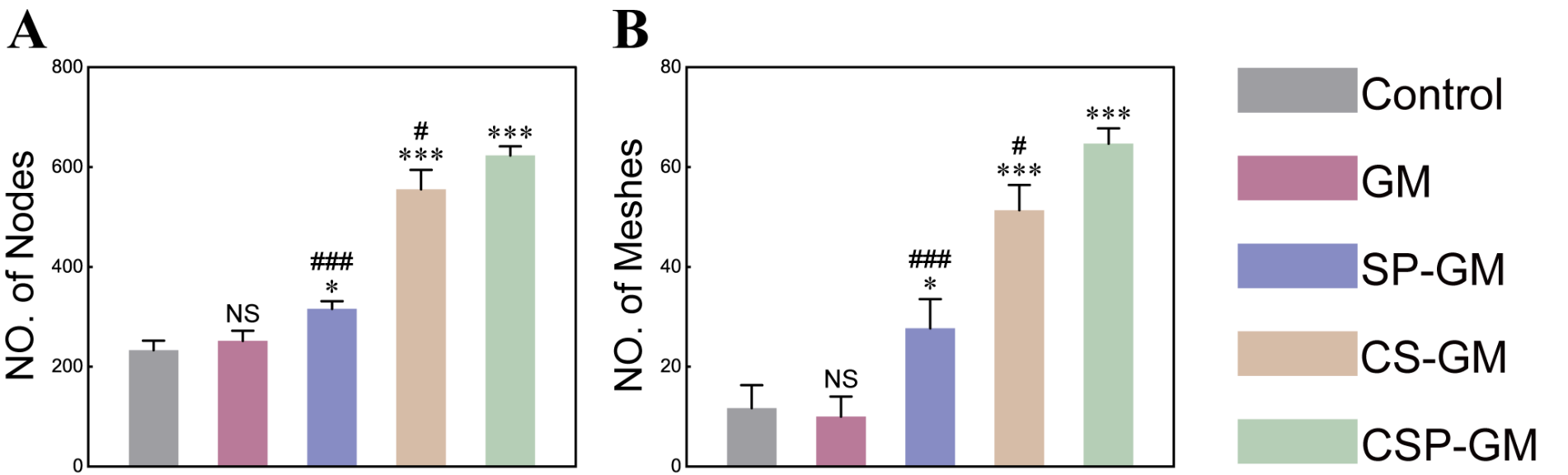
**

**Figure S15.** Quantitative analysis of A) number of nodes (n=3), B) number of meshes (n=3). NS: no significance, ^*^*P* < 0.05, ^**^*P* < 0.01, and ^***^*P* < 0.001 compared with the Control group; ^#^*P* < 0.05, ^##^*P* < 0.01, and ^###^*P* < 0.001 compared with the CSP-GM group.


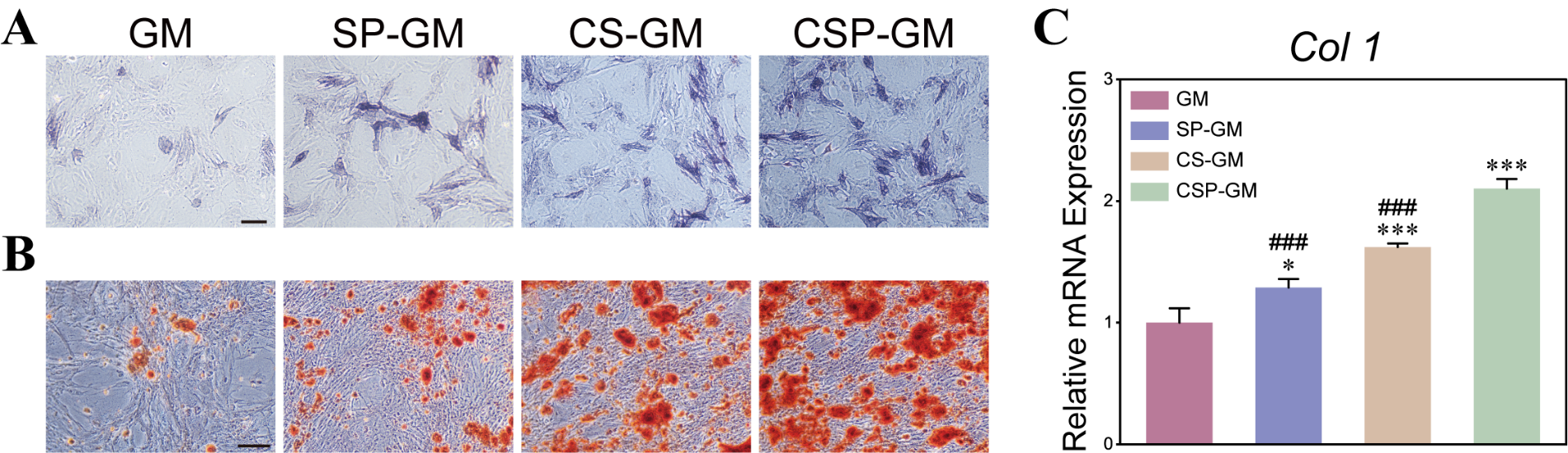


**Figure S16.** A) ALP staining at 7 days of culturing with microspheres after osteogenic induction (scale bar, 100 μm). B) ARS staining at 7 days of culturing with microspheres after osteogenic induction (scale bar, 300 μm). C) Relative mRNA expression of *Col 1* at 7 days detected by qRT-PCR (n=3). NS: no significance, ^*^*P* < 0.05, ^**^*P* < 0.01, and ^***^*P* < 0.001 compared with the GM group; ^#^*P* < 0.05, ^##^*P* < 0.01, and ^###^*P* < 0.001 compared with the CSP-GM group.

**
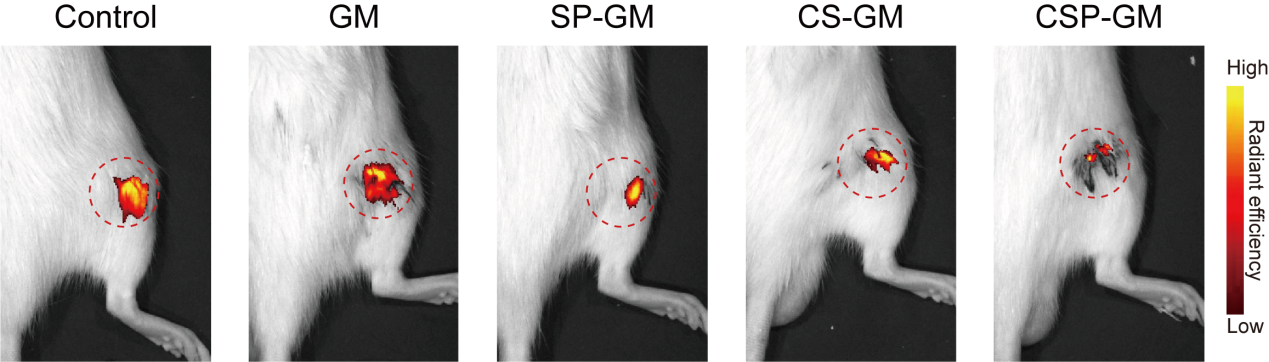
Figure S17.** In vivo fluorescence images of ROS in bone defects at 1-week post-operation.

**
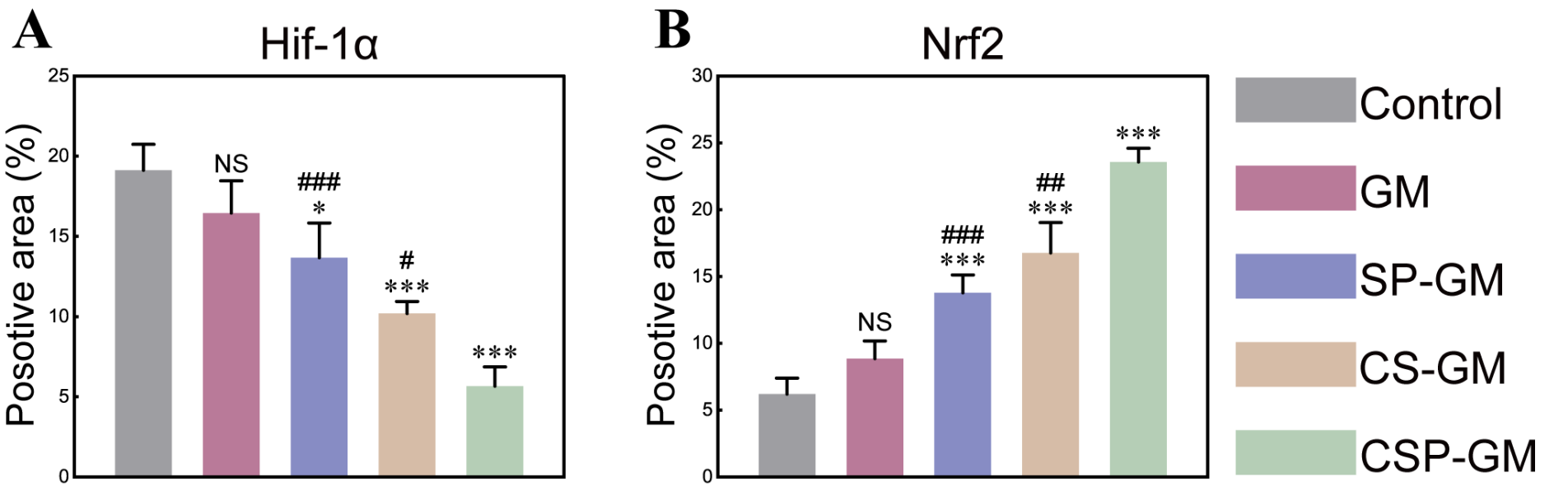
**

**Figure S18. In vivo hypoxia-alleviation and antioxidant activities.** Quantitative analysis of positive areas of A) Hif-1α and B) Nrf2 at 2 weeks postoperative (n=3). NS: no significance, ^*^*P* < 0.05, ^**^*P* < 0.01, and ^***^*P* < 0.001 compared with the Control group; ^#^*P* < 0.05, ^##^*P* < 0.01, and ^###^*P* < 0.001 compared with the CSP-GM group.

**
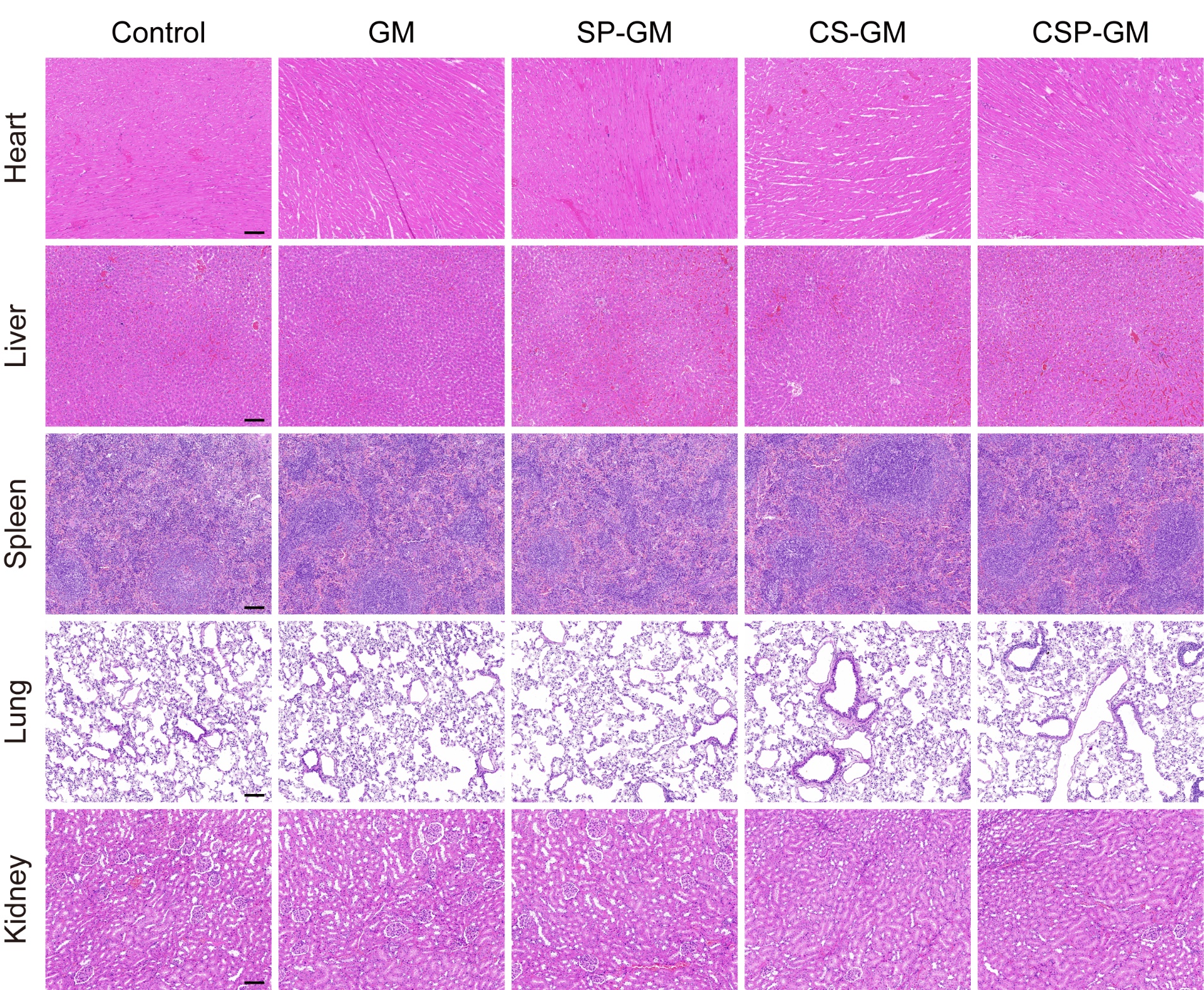
**

**Figure S19.** H&E staining of major organs, including the heart, liver, spleen, lung and kidney at 4 weeks post-operation. Scale bar, 200μm.

**References**

1. J. Khodaveisi, H. Banejad, A. Afkhami, E. Olyaie, S. Lashgari, R. Dashti, [*J. Hazard Mater*](https://www.ablesci.com/journal/detail?id=DY7Nvp) **2011**, 192, 1437.
2. J. Huang, S. Wang, X. Wang, J. Zhu, Z. Wang, X. Zhang, K. Cai, J. Zhang, *Acta biomater* **2022**, 152, 171.
